# Dietary patterns and cardiometabolic diseases in 0.5 million Chinese adults: a 10-year cohort study

**DOI:** 10.1186/s12937-021-00730-4

**Published:** 2021-09-03

**Authors:** Chenxi Qin, Jun Lv, Canqing Yu, Yu Guo, Zheng Bian, Meng Gao, Huaidong Du, Ling Yang, Yiping Chen, Leijia Shen, Songgen Zhou, Junshi Chen, Zhengming Chen, Liming Li

**Affiliations:** 1 Department of Epidemiology and Biostatistics, School of Public Health, Peking University Health Science Center, 38 Xueyuan Road, Beijing, 100191 China; 2 Nuffield Department of Population Health, Clinical Trial Service Unit and Epidemiological Studies Unit (CTSU), University of Oxford, Oxford, UK; 3 Key Laboratory of Molecular Cardiovascular Sciences (Peking University), Ministry of Education, Beijing, China; 4 Peking University Institute of Environmental Medicine, Beijing, China; 5 Chinese Academy of Medical Sciences, Beijing, China; 6 Medical Research Council Population Health Research Unit at the University of Oxford, Oxford, UK; 7 The Second hospital of Tongxiang, Tongxiang, Zhejiang Province China; 8 Wuzhen Town Health Centres, Tongxiang, Zhejiang Province China; 9 China National Center for Food Safety Risk Assessment, Beijing, China

**Keywords:** Dietary pattern, Cardiovascular disease, Diabetes, Cohort

## Abstract

**Background:**

The effect of the overall diet quality on cardiometabolic diseases has been well studied in the Western population. However, evidence is still in need regarding dietary patterns depicting unique Chinese dietary habits and their associations with cardiometabolic diseases.

**Methods:**

A prospective cohort recruited around 0.5 million Chinese residents aged 30–79 years from 10 diverse survey sites during 2004–08. Dietary patterns were obtained using factor analysis based on the habitual consumption of 12 food groups collected at baseline. Among 477,465 eligible participants free of prior heart disease, stroke and cancer, linkages to multiple registries and health insurance database recorded 137,715 cardiovascular diseases (CVD) and 17,412 diabetes cases (among 451,846 non-diabetic participants) until 31 December 2017. Adjusted hazard ratios (HRs) were estimated to compare the risks of cardiometabolic diseases across quintiles of dietary pattern scores using the Cox regression.

**Results:**

Two dietary patterns were derived: the traditional northern pattern, characterised by wheat, other staples, egg and dairy products; and the modern pattern, featured with fresh fruit, meat, poultry, fish, dairy products and soybean. Adherence to either dietary pattern was associated with lower risks of major cardiometabolic diseases in a dose-response relationship way. After multivariate adjustment, participants adhering to the traditional northern pattern the most had an 8% (95%CI: 5–11%) lower risk of CVD in comparison with those adhering the least. Corresponding risk reductions were 12% (11–32%) for haemorrhagic stroke (HS), 14% (8–19%) for ischaemic stroke (IS), and 15% (6–24%) for diabetes, respectively. When comparing extreme quintiles of the modern pattern, the adjusted HR of HS was 0.67 (95%CI: 0.59–0.77). Corresponding HRs were 0.89 (0.86–0.92) for CVD, 0.88 (0.77–0.99) for MCE, 0.85 (0.80–0.89) for IS, and 0.89 (0.81, 0.97) for diabetes.

**Conclusion:**

Among Chinese adults, both traditional northern and modern dietary patterns were associated with lower risks of cardiovascular disease and diabetes beyond other risk factors.

**Supplementary Information:**

The online version contains supplementary material available at 10.1186/s12937-021-00730-4.

## Introduction

Cardiometabolic diseases, including cardiovascular disease (CVD) and diabetes, were accountable for 34% of 57 million deaths globally and 45% of 10 million China’s deaths in 2016 [1]. Diet is a crucial and modifiable lifestyle risk factor in cardiometabolic diseases pathogenesis. A dietary pattern can depict the overall diet quality without emphasising particular nutrients and considering nutrient interaction effects. Priori diet indices like the Mediterranean diet and Dietary Approaches to Stop Hypertension (DASH) were designed based on hypotheses and have been proved to prevent CVD directly in the high-risk group or through reducing blood pressure [2–4], as well as diabetes risk [3, 5, 6]. Previous studies using this approach have identified healthy dietary patterns, characterised with high intakes of vegetable, fruit, fish, poultry, whole grain and low-fat dairy products, were associated with decreased CVD mortality [7, 8].

Despite that healthy dietary indices have been reported and applied in some dietary guidelines, healthy dietary patterns identified via a posterior way could reflect food culture, diversity and accessibility, and disease pattern in different populations. Unlike in western countries, a higher prevalence and incidence of haemorrhagic stroke were reported in the Chinese population [9] and its relationships to healthy dietary patterns were inconsistent, lacking sufficient cases, and mostly from the white population [10–12]. Therefore, it is critical to describe Chinese dietary patterns and estimate their effects on cardiometabolic diseases, especially with haemorrhagic stroke.

Our study aimed to identify major dietary patterns in the Chinese population and investigate their associations with cardiometabolic diseases in the China Kadoorie Biobank (CKB).

## Methods

### Study population

The CKB study is an ongoing prospective cohort study involving around half a million Chinese adults from five rural and five urban survey sites. In brief, the baseline study recruited 512,726 participants aged 30–79 years between June 2004 and July 2008 and followed up the morbidity and mortality ever since. More details about CKB have been published previously [13, 14]. After completing the baseline survey, CKB randomly selected 5% of total participants and revisited them in the first and second resurveys (2008 and 2013–2014, respectively). The present study excluded participants with prior heart disease, stroke or transient ischaemic attack, or cancer (*n* = 25,514), missing body mass index (BMI, *n* = 2), illogical censoring date (*n* = 1), or daily energy intake outside the range of 2–99 percentiles, leaving 477,465 eligible participants. Individuals with diagnosed or screened diabetes were further excluded in analyses related to diabetes (*n* = 451,846).

### Dietary intake assessment

At baseline, a qualitative food frequency questionnaire (FFQ) was administered to collect the habitual consumption over the past 12 months. The FFQ contained 12 common food groups recommended by the Chinese Dietary Guidelines, including rice, wheat products, other staples (such as millet, corn and sorghum), meat, poultry, fish, eggs, dairy products, fresh vegetables, preserved vegetables, fresh fruit and soybean products [15]. Five frequency levels were alternative: never or rarely, monthly, 1–3 days/week, 4–6 days/week and daily, which were recoded into 0, 0.5, 2, 5, 7 days/week, respectively. The intake amount was further asked in the second resurvey. Assuming that the amount does not change materially over time, the average amount of each frequency level at baseline was predicted through modelling the amount by frequency level, survey site, sex, and age (10-year interval) using the second resurvey data. Multiplying the frequency level by the predictive amount produced the weekly amount [16]. The reproducibility and relative validity of FFQsin the baseline and second resurvey were assessed in 2015–2016. The average weighted kappa coefficients for qualitative FFQ were 0.77 and 0.74, respectively (data not published). The average Spearman correlation coefficients for quantitative FFQ were 0.36 and 0.35, respectively, indicating moderate reproducibility and relative validity. But the relative validity of other staples and fresh vegetables were below 0.2 in the quantitative FFQ of second resurvey.

### Covariates evaluations

The baseline survey also collected information on socio-demographic characteristics (age at recruitment, gender, and education level), lifestyle behaviours (smoking, alcohol drinking, spicy food consumption, and physical activity), medical history (diagnosed hypertension and diagnosed diabetes), family history of CVD and diabetes, and menopause status (only for women). Body weight, height, waist circumference, blood pressure, random blood glucose, and fasting blood glucose were measured or tested by skilled staff following standard protocols.

The physical activity level was quantified by adding up products of average hours spent on each activity and its corresponding metabolic equivalence task (MET) value [17]. Hypertension was considered if a participant reported a diagnosis, took antihypertensive drugs before measuring blood pressure, or had a measured systolic blood pressure ≥ 140 mmHg or diastolic blood pressure ≥ 90 mmHg. Diabetes existed if a participant reported a diagnosis, had a fasting blood glucose level ≥ 7.0 mmol/L or a random blood glucose level ≥ 11.1 mmol/L [18]. A participant had a family history of CVD if at least one of his/her biological parents or siblings had a heart attack or stroke. The family history of diabetes was calculated in the same way.

### Endpoint ascertainment

The CKB study linked to local disease and death registries, checked against the national health insurance database, and periodically carried out active follow-ups to monitor participants’ vital signs and disease status. Clinicians, unaware of baseline characteristics, coded diseases based on the International Classification of Diseases, 10th Revision (ICD-10). Primary endpoints included CVD (I00-I99), major coronary events (MCE, including nonfatal acute myocardial infarction [AMI, I21] and fatal ischaemic heart disease [IHD, I20-I25]), haemorrhagic stroke (HS, I61), ischaemic stroke (IS, I63), and diabetes (E10-E14). Secondary endpoints included IHD, AMI, cerebrovascular disease (CBD, I60-I69), total stroke (I60, I61, I63, I64), subarachnoid stroke (I60), and pulmonary heart disease (I26-I27).

### Statistical analysis

Dietary patterns were derived from 12 food groups using factor analysis with the principal component method. Factors were retained if they had an eigenvalue > 1 and explained ≥10% of the total variance. Food groups with a factor loading > 0.4 represented characteristics of a dietary pattern. Dietary pattern scores, i.e. factor scores, were divided into quintiles and treated as exposures in further analyses.

Age-, sex-, and site-adjusted means or proportions were compared across quintiles of dietary patterns. Cox proportional hazard models, stratified by survey sites and age-at-risk (5-year groups), estimated the hazard ratios (HR) and 95% confidence intervals (CIs) relating dietary patterns to diseases. Model 1 adjusted for age at recruitment, sex, and education level (no formal school, primary school, middle school, high school, college, or university or higher). Model 2 additionally adjusted for smoking (never or occasional; former; current smoking with 1–14, 15–24, or ≥ 25 cigarettes/day), alcohol consumption (not weekly; ex-regular; not daily; daily consuming 1–15, 15–29, 30–59 or ≥ 60 g), physical activity level (MET-hours/day), the average daily energy intake (kcal), family history of CVD or diabetes (yes or no), BMI (kg/m^2^), and waist circumference (cm). Model 3 additionally adjusted for diabetes, antihypertensive drugs use, and systolic blood pressure (mmHg). Person-years at risk elapsed from the completion of the baseline survey to the diagnosis of CVD, death, loss to follow-up or 31 December 2017, whichever came first. *P* values of interaction terms between dietary pattern and endpoints indicated no violation of proportional hazard assumptions. The median value was assigned to each quintile and treated as the exposure to test the linear trend.

Cases occurring in the first 2 years of follow-up were further excluded to minimise the possibility of reverse causality. Aspirin and statin use were additionally adjusted in sensitivity analyses. Dietary patterns exclusion of other staples and fresh vegetables were generated and used in multivariate Cox models to yield the hazards of primary and secondary endpoints. Joint effects of dietary patterns on cardiometabolic diseases were estimated using participants with the lowest quintiles of both dietary patterns as the reference group, and multiplicative interactions were tested by joint tests. Adjusted HRs of each 2 increment score for MCE, HS, IS, and diabetes were compared across stratum of baseline covariates to judge whether modifying effects existed, and Wald chi-square tests were performed to evaluate the heterogeneity. The significant level was set at 2-tailed *P* < 0.05. All statistical analyses were done using SAS 9.4.

## Results

Overall, 59.1% of 477,465 participants were women, 43.0% were urban residents, and the average age at recruitment was 51.1 years (standard deviation: 10.5 years). Two factors were retained, explaining 42.2% of the total variation (Supplemental Table 1). The first factor, labelled as ‘traditional northern’, was featured with high intakes of wheat, other staples and egg, moderate intake of dairy products, and low intakes of rice and preserved vegetables. The second factor, labelled as ‘modern’, had high loadings on fresh fruit, meat, poultry, fish, dairy products and soybean. The average intake of each food group across quintiles was listed in Supplemental Table 2.

Participants with a higher score of either dietary pattern tended to be male, well-educated and non-current smokers, and had lower blood pressure (Table 1). Those sticking to the traditional northern dietary pattern were more likely to be rural residents but less likely to be weekly alcohol consumers. Participants adopting the modern dietary pattern tended to reside in urban areas, be younger and physically inactive, and have a family history of CVD and diabetes.

**Table 1:** Baseline characteristics of participants by quintiles dietary pattern scores (*n* = 477,465)

Baseline characteristics	Traditional northern dietary pattern			Modern dietary pattern		
	Q1	Q3	Q5	Q1	Q3	Q5
Age, mean (SD), y	53.4 (9.8)	49.5 (10.7)	51.6 (10.5)	55.6 (10.8)	51.3 (10.3)	47.9 (10.5)
Women, %	69.7	57.3	50.7	77.8	58.9	45.4
Urban residents, %	45.8	52.7	19.1	6.7	33.9	89.2
Highest education level, %						
Primary school and below	65.4	46.2	27.3	64.8	49.4	24.3
Middle or high school	34.1	51.1	64.9	34.9	48.9	66.0
Current smokers, %						
Women	1.2	1.0	0.4	1.0	0.9	0.4
Men	74.4	68.7	59.0	68.3	69.8	61.5
Weekly drinkers, %						
Women	1.4	1.1	1.2	0.9	1.1	1.8
Men	38.2	31.9	25.5	21.5	32.8	32.6
Physical activity, mean (SD), MET h/d	22.9 (15.0)	20.8 (12.3)	20.7 (14.9)	22.0 (14.2)	21.8 (14.5)	20.0 (11.7)
BMI, mean (SD), kg/m^2^	23.6 (3.3)	23.8 (3.2)	23.4 (3.4)	23.3 (3.5)	23.6 (3.3)	23.8 (3.3)
Waist circumference, mean (SD), cm	80.3 (9.3)	81.0 (9.5)	80.0 (9.6)	79.5 (9.6)	80.5 (9.4)	81.2 (9.8)
Systolic blood pressure, mean (SD), mmHg	132.5 (21.4)	131.1 (20.3)	127.2 (20.9)	131.4 (22.1)	130.8 (20.9)	129.0 (19.8)
Diastolic blood pressure, mean (SD), mmHg	78.9 (10.8)	78.1 (10.9)	75.9 (11.2)	78.0 (11.3)	77.9 (11.0)	77.2 (11.0)
Hypertension, %	34.4	33.1	25.9	32.1	31.8	29.8
Diabetes, %	3.5	4.6	5.7	3.9	4.6	4.8
Medication in diagnosed hypertension, %						
Antihypertensive medication	41.1	51.7	49.9	43.4	50.4	53.3
Statin	1.6	1.5	0.9	1.1	1.1	1.2
Aspirin	3.0	5.5	4.5	3.3	4.9	5.1
Family history, %						
CVD	17.6	18.7	20.6	16.1	18.8	20.4
Diabetes*	4.0	5.4	6.7	3.9	5.1	7.1

All variables were standardised for age, sex and survey sites according to eligible participants, as appropriate

MET: metabolic equivalent; BMI: body mass index. SD: standard deviation

* Adjusted proportions were estimated in the non-diabetic population

After a median of 10.5 years (4.6 million person-years) of follow-up, the CKB study tracked 137,715 CVD (including 8870 MCE, 9758 HS and 42,667 IS cases) and 17,412 diabetes cases (in the non-diabetic population). The traditional northern dietary pattern was inversely associated with major cardiometabolic diseases (Table 2 and Supplemental Table 3). Compared with the lowest quintile, the highest quintile the traditional northern dietary pattern was associated with a multivariate-adjusted HR of 0.92 (95%CI, 0.89–0.95) for CVD (*P* for linear trend: < 0.001). Corresponding HRs (95%CIs) were 0.92 (0.81, 1.05) for MCE, 0.78 (0.68–0.89) for HS, 0.86 (0.81–0.92) for IS, and 0.85 (0.76–0.94) for diabetes (*P* values for linear trends: MCE, 0.118; other endpoints, < 0.001).

**Table 2:** Hazard ratios of cardiometabolic diseases by quintiles of the traditional northern dietary pattern among 477,465 participants

Endpoints	Traditional northern dietary pattern					*P*_*trend*_
	Q1	Q2	Q3	Q4	Q5	
CVD						
Cases	25,118	27,083	26,571	30,448	28,495	
Incidence density (1/1000 PYs)	26.1	25.6	25.5	24.4	23.1	
Model 1	1.00 (Ref.)	0.98 (0.96, 0.99)	0.99 (0.97, 1.01)	0.96 (0.93, 0.98)	0.90 (0.87, 0.93)	< 0.001
Model 2	1.00 (Ref.)	0.97 (0.95, 0.98)	0.96 (0.94, 0.98)	0.94 (0.91, 0.96)	0.90 (0.87, 0.93)	< 0.001
Model 3	1.00 (Ref.)	0.97 (0.96, 0.99)	0.96 (0.94, 0.98)	0.94 (0.92, 0.97)	0.92 (0.89, 0.95)	< 0.001
MCE						
Cases	966	1596	1665	2277	2366	
Incidence density (1/1000 PYs)	1.0	0.9	0.9	0.8	0.8	
Model 1	1.00 (Ref.)	1.01 (0.93, 1.11)	0.98 (0.90, 1.07)	0.90 (0.80, 1.00)	0.88 (0.77, 1.00)	0.017
Model 2	1.00 (Ref.)	1.00 (0.92, 1.09)	0.97 (0.88, 1.06)	0.91 (0.81, 1.01)	0.92 (0.80, 1.04)	0.121
Model 3	1.00 (Ref.)	1.01 (0.93, 1.10)	0.96 (0.87, 1.05)	0.90 (0.80, 1.00)	0.92 (0.81, 1.05)	0.118
HS						
Cases	1654	2085	1749	2132	2138	
Incidence density (1/1000 PYs)	1.5	1.4	1.2	1.1	0.8	
Model 1	1.00 (Ref.)	0.95 (0.89, 1.02)	0.86 (0.79, 0.93)	0.81 (0.73, 0.90)	0.62 (0.54, 0.71)	< 0.001
Model 2	1.00 (Ref.)	0.95 (0.89, 1.03)	0.87 (0.81, 0.94)	0.85 (0.76, 0.94)	0.68 (0.59, 0.78)	< 0.001
Model 3	1.00 (Ref.)	1.00 (0.93, 1.07)	0.89 (0.82, 0.96)	0.89 (0.80, 0.98)	0.78 (0.68, 0.89)	< 0.001
IS						
Cases	5883	6947	8328	10,708	10,801	
Incidence density (1/1000 PYs)	6.5	6.4	6.3	5.6	5.1	
Model 1	1.00 (Ref.)	1.01 (0.97, 1.05)	1.01 (0.97, 1.05)	0.92 (0.88, 0.96)	0.84 (0.79, 0.89)	< 0.001
Model 2	1.00 (Ref.)	1.00 (0.96, 1.04)	0.98 (0.94, 1.02)	0.91 (0.86, 0.95)	0.84 (0.79, 0.89)	< 0.001
Model 3	1.00 (Ref.)	1.01 (0.97, 1.04)	0.97 (0.94, 1.01)	0.91 (0.87, 0.96)	0.86 (0.81, 0.92)	< 0.001
Diabetes						
Cases	5433	4141	3619	2493	1726	
Incidence density (1/1000 PYs)	3.3	3.1	3.2	2.8	2.7	
Model 1	1.00 (Ref.)	0.93 (0.89, 0.97)	1.00 (0.95, 1.05)	0.86 (0.80, 0.92)	0.82 (0.74, 0.92)	< 0.001
Model 2	1.00 (Ref.)	0.91 (0.87, 0.95)	0.92 (0.88, 0.97)	0.80 (0.74, 0.86)	0.82 (0.74, 0.92)	< 0.001
Model 3	1.00 (Ref.)	0.91 (0.87, 0.96)	0.93 (0.88, 0.98)	0.81 (0.75, 0.87)	0.85 (0.76, 0.94)	< 0.001

Incidence density was adjusted for age at recruitment, sex and survey sites. Hazard ratios (HRs) were estimated using Cox models with stratification on survey sites and age-at-risk (5-year groups). Model 1 was adjusted for sex, age at recruitment, education level. Model 2 was additionally adjusted for smoking, alcohol consumption, physical activity, the average daily energy intake, spicy food, family history of CVD or diabetes, body mass index, and waist circumference. Model 3 was additionally adjusted for prevalent diabetes, antihypertensive drugs use, and systolic blood pressure. Tests for linear trend were conducted by assigning the median value to each quintile and modelling it as a continuous variable in the Cox model

CVD: cardiovascular disease. MCE: major coronary events. HS: haemorrhagic stroke. IS: ischaemic stroke. PY: person year

* Analyses were performed among 451,846 diabetic participants

Likewise, inverse associations were observed between the modern dietary pattern and major cardiometabolic diseases (Table 3 and Supplemental Table 4). Adjusted HRs of comparisons of the highest with the lowest quintile were 0.89 (95%CI, 0.86–0.92) for CVD, 0.88 (0.77–0.99) for MCE, 0.67 (0.59–0.77) for HS, and 0.85 (0.80–0.89) for IS (all *P* values for linear trend: ≤0.001). Participants with the most adherence had an 11% (95%CI, 3–19%) lower risk of diabetes than those with the lowest adherence (*P* for linear trend: 0.015).

**Table 3:** Hazard ratios of cardiometabolic diseases by quintiles of the modern dietary pattern among 477,465 participants

Endpoints	Modern dietary pattern					*P*_*trend*_
	Q1	Q2	Q3	Q4	Q5	
CVD						
Cases	30,630	28,772	27,420	26,337	24,556	
Incidence density (1/1000 PYs)	25.4	25.1	25.3	25.2	23.8	
Model 1	1.00 (Ref.)	0.99 (0.98, 1.01)	0.99 (0.97, 1.01)	0.99 (0.97, 1.01)	0.95 (0.93, 0.97)	< 0.001
Model 2	1.00 (Ref.)	0.97 (0.96, 0.99)	0.95 (0.93, 0.97)	0.92 (0.89, 0.94)	0.87 (0.84, 0.90)	< 0.001
Model 3	1.00 (Ref.)	0.98 (0.97, 1.00)	0.96 (0.94, 0.98)	0.93 (0.91, 0.96)	0.89 (0.86, 0.92)	< 0.001
MCE						
Cases	2193	1712	1618	1637	1710	
Incidence density (1/1000 PYs)	1.0	0.9	0.9	0.8	0.7	
Model 1	1.00 (Ref.)	0.92 (0.86, 0.99)	0.92 (0.86, 0.99)	0.90 (0.83, 0.97)	0.81 (0.74, 0.88)	< 0.001
Model 2	1.00 (Ref.)	0.93 (0.87, 1.00)	0.92 (0.85, 1.00)	0.90 (0.81, 0.99)	0.85 (0.75, 0.96)	0.014
Model 3	1.00 (Ref.)	0.94 (0.88, 1.01)	0.93 (0.86, 1.01)	0.91 (0.83, 1.00)	0.88 (0.77, 0.99)	0.046
HS						
Cases	2888	2393	1993	1473	1011	
Incidence density (1/1000 PYs)	1.5	1.4	1.2	1.1	0.8	
Model 1	1.00 (Ref.)	0.93 (0.88, 0.99)	0.88 (0.82, 0.93)	0.78 (0.73, 0.84)	0.61 (0.56, 0.67)	< 0.001
Model 2	1.00 (Ref.)	0.93 (0.87, 0.98)	0.86 (0.80, 0.93)	0.76 (0.69, 0.84)	0.60 (0.53, 0.69)	< 0.001
Model 3	1.00 (Ref.)	0.96 (0.90, 1.02)	0.90 (0.84, 0.97)	0.83 (0.75, 0.91)	0.67 (0.59, 0.77)	< 0.001
IS						
Cases	9729	7853	7720	9055	8310	
Incidence density (1/1000 PYs)	6.1	6.1	6.0	6.2	5.4	
Model 1	1.00 (Ref.)	1.00 (0.97, 1.03)	0.99 (0.96, 1.03)	1.02 (0.98, 1.06)	0.90 (0.87, 0.94)	< 0.001
Model 2	1.00 (Ref.)	0.97 (0.94, 1.01)	0.94 (0.90, 0.97)	0.93 (0.89, 0.98)	0.82 (0.77, 0.86)	< 0.001
Model 3	1.00 (Ref.)	0.99 (0.96, 1.02)	0.95 (0.92, 0.99)	0.95 (0.91, 1.00)	0.85 (0.80, 0.89)	< 0.001
Diabetes						
Cases	3014	3737	3931	3625	3105	
Incidence density (1/1000 PYs)	2.9	2.8	3.0	3.1	3.2	
Model 1	1.00 (Ref.)	0.97 (0.92, 1.02)	1.03 (0.98, 1.08)	1.07 (1.01, 1.13)	1.12 (1.05, 1.20)	< 0.001
Model 2	1.00 (Ref.)	0.93 (0.88, 0.98)	0.93 (0.87, 0.98)	0.88 (0.82, 0.94)	0.88 (0.80, 0.96)	0.009
Model 3	1.00 (Ref.)	0.93 (0.88, 0.98)	0.92 (0.87, 0.98)	0.88 (0.82, 0.94)	0.89 (0.81, 0.97)	0.015

Incidence density was adjusted for age at recruitment, sex and survey sites. Hazard ratios (HRs) were estimated using Cox models with stratification on survey sites and age-at-risk (5-year groups). Model 1 was adjusted for sex, age at recruitment, education level. Model 2 was additionally adjusted for smoking, alcohol consumption, physical activity, the average daily energy intake, spicy food, family history of CVD or diabetes, body mass index, and waist circumference. Model 3 was additionally adjusted for prevalent diabetes, antihypertensive drugs use, and systolic blood pressure. Tests for linear trend were conducted by assigning the median value to each quintile and modelling it as a continuous variable in the Cox model

CVD: cardiovascular disease. MCE: major coronary events. HS: haemorrhagic stroke. IS: ischaemic stroke. PY: person year

* Analyses were performed among 451,846 diabetic participants

Results did not materially change after excluding endpoints occurring in the first 2-year follow-up or additional adjustment for statin or aspirin use (Supplemental Table 5–7). Dietary patterns without other staples and fresh vegetables shared similar characteristics with those from primary analyses (Supplemental Table 8), and their associations with all endpoints remained robust (Supplemental Table 9).

**Fig. 1:**
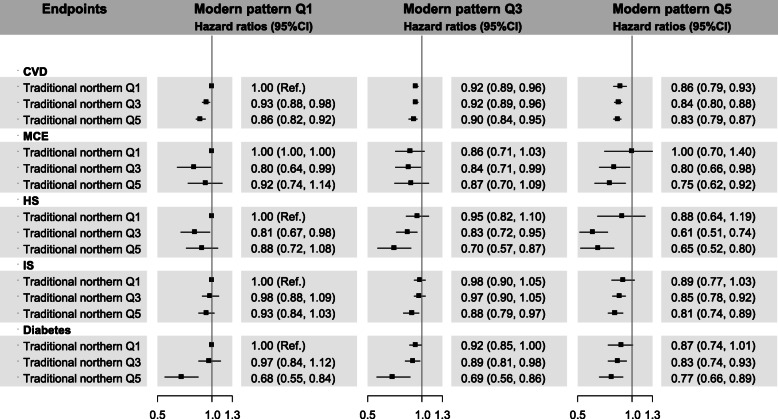
Hazard ratios of selected cardiovascular diseases according to the joint classification of dietary patterns. Hazard ratios (HRs) were estimated using Cox models with stratification on survey sites and age-at-risk (5-year groups), and adjustment for sex, age at recruitment, education level, smoking, alcohol consumption, physical activity, the average daily energy intake, spicy food, family history of CVD or diabetes, body mass index, waist circumference, prevalent diabetes, antihypertensive drugs use, and systolic blood pressure. CVD: cardiovascular disease. MCE: major coronary events. HS: haemorrhagic stroke. IS: ischaemic stroke. *P* values for interaction of two dietary patterns were < 0.001, 0.818, 0.012, 0.261, and 0.142, respectively

Compared with non-adherence to either dietary pattern, simultaneous adherence to both patterns was associated with lower CVD risk (0.83, 0.79–0.87) and HS risk (0.65, 0.52–0.80) (*P* for interaction: < 0.001 and 0.012, respectively) (Fig. 1 and Supplemental Table 10). Participants complying with both patterns also had lower risks of MCE (0.75, 0.62–0.92), IS (0.81, 0.74–0.89), and diabetes (0.77, 0.66–0.89), but multiplicative effects were insignificant.

Adjusted HRs for CVD, MCE, HS, IS, and diabetes were similar across stratum of most potential effect modifiers, though heterogeneity existed (Supplemental Fig. 1–4). For the traditional northern pattern, risks of HS and IS appeared stronger in women compared with men (*P* for interaction: 0.032 and 0.020, respectively). Meantime, the magnitude of associations between the traditional northern pattern and HS and diabetes were more pronounced in hypertensive individuals than non-hypertensive ones (both *P* values for interaction: < 0.001). Women adhering to the modern pattern has a greater decrease in the MCE risk than men (*P* for interaction: < 0.001). Risk reductions of HS and IS attributed to the modern pattern were lower in diabetic patients than non-diabetic ones (P for interaction: 0.027 and < 0.001, respectively).

## Discussion

The present study obtained a traditional northern dietary pattern, characterised with consuming more wheat, other staples and eggs, moderate dairy products, and less rice and preserved vegetables; and a modern dietary pattern, featured with fresh fruit, meat, poultry, fish, dairy products and soybean, from ~ 0.5 million Chinese adults. Adherence to either dietary pattern was associated with reduced CVD, MCE, HS, IS and diabetes risks, independent of other established risk factors. In particular, participants complying with both dietary pattern had a 17% decreased CVD risk and a decreased 35% HS risk.

The present study showed that compliance with the traditional northern dietary pattern was associated with reduced risks of major CVD and diabetes. The traditional northern dietary pattern showed positive loadings of eggs and dairy products but negative loadings on meat and fish, suggesting that the traditional northern pattern had similarities with the vegetarian diet [19]. In a recent meta-analysis pooling estimates from 8 prospective cohort studies, the relative risk of the vegetarian diet was 0.93 (95%CI, 0.86–1.00) for CVD, 0.75 (0.68–0.82) for IHD, and 0.93 (0.78–1.10) for CBD [20]. In the EPIC-Oxford cohort, vegetarians had a 22% (HR, 0.78; 95%CI 0.70–0.87) lower rate of IHD compared with meat-eaters [12]. Our findings of CVD and stroke were in line with previous results, though the reduced MCE risk was insignificant. The CVD risk gap may result from different plant-based groups and food evaluation methods. Pooled odds ratio from a meta-analysis of 14 studies for the vegetarian diet and diabetes was 0.73 (0.61–0.87) [21]. And a prospective Chinese cohort reported consistent results that vegetarians had a 35% lower rate of diabetes; moreover, converting from a non-vegetarian diet to a vegetarian one was associated with a 53% lower diabetes risk [22]. These consistent results suggested that a vegetarian-like diet may prevent CVD and diabetes beyond other established risk factors.

The vegetarian diet was reported to decelerate CVD pathological process through reducing oxidative stress, improving endothelial function, and decreasing systemic inflammation, suggesting these possible mediating pathways [23]. Likewise, prominent foods in the traditional northern dietary pattern also support the potential protective effects above. Consistent findings have demonstrated whole grain was in association with lower risks of CVD and diabetes [24, 25]. Whole grain contains abundant dietary fibre that can decrease body weight, blood pressure and total cholesterol, alleviate systemic inflammation [26, 27]. Egg intake was reported to be associated with a reduced CVD risk in the Chinese population [28], yet a null association with diabetes was found [25]. The CVD risk associated with dairy products was null [29], but the associated diabetes risk was lower in the Asian population [25]. Given negative loadings on animal foods and soybean products, eggs and dairy products in the traditional northern pattern may exert more significant health effects via supplying nutrients like protein and calcium. Besides, a lower intake of preserved vegetables accompanied by reduced salt intake may partially explain the potential atherosclerotic benefits as well [30].

In this study, individuals adhering to the modern dietary pattern showed a reduction in CVD and diabetes risks. A fruit-rich pattern was associated with decreased mortality from CVD (0.89, 0.81–0.99), stroke (0.79, 0.69–0.91), and diabetes (0.51, 0.39–0.65) when comparing the extreme quartiles in Shanghai Women’s Health Study [31]. The Singapore Chinese Health Study observed individuals sticking to a vegetable-, fruit-, and soy-rich pattern had a 37% (28–44%) decreased CVD mortality [8]. The discrepancy of effect sizes could result from the data-driven nature of posterior dietary patterns.

The modern dietary pattern had some high-loading components such as fruit and fish, which were also the main contributors to the Mediterranean and DASH diet. Daily fresh fruit consumption was in association with lower risks of cardiometabolic diseases in CKB participants [32, 33]. Fruit is a rich source of numerous nutrients and phytochemicals, such as dietary fibre, potassium, vitamins and antioxidants, thus improving lipid profiles and insulin sensitivity, reducing blood pressure, regulating haemostasis, and neutralising oxidative reaction [34]. Fish supplies n-3 polyunsaturated fatty acids that may also prevent CVD through the aforementioned physiological mechanisms as well as the antiarrhythmic process [35]. Even though pooled associations with cardiometabolic diseases were insignificant and heterogeneous, soy, poultry and dairy products are biologically plausible to benefit vascular health owing to nutrients like proteins, isoflavones, and unsaturated fatty acids [29, 36, 37]. And these food groups were usually regarded as healthy alternatives in dietary indices [2, 4]. Notably, the modern dietary pattern had a high loading of meat. Positive associations between cardiometabolic diseases and processed meat were observed in numerous studies, and current evidence about red meat only support moderate intake (100–200 g/week) [38]. Although participants complying with the modern pattern consumed 1100 g per week in the present study, deleterious effects of meat may be counteracted by healthy food groups in the modern dietary pattern.

Strengths of the present study included a large sample size, a broader age range, careful adjustment for established and potential risk factors, and investigating the effects of dietary patterns on haemorrhagic stroke. Yet there are some limitations to consider. The FFQ used in the present study was self-reported, recall bias could lead to potential misclassification and affect factor loadings in dietary patterns. We used the second resurvey data to estimate the amount of each food group based on the assumption that participants would not alter their habitual consuming amounts between two surveys. The present study only used 12 major food groups and beverages to construct dietary patterns. However, some other dietary habits were reported to cause cardiometabolic diseases, such as deep-fried or sweet foods. The second resurvey found that a 97.1, 96.0, 92.8, and 99.5% majority consumed less than one day per week of carbonated drinks, other soft drinks, deep-fried foods and Western-type fast foods, respectively. Incorporating these foods may not change the main features of dietary patterns since they can only contribute limited proportion of total variability. Finally, residual confounding could still bias the observed associations.

## Conclusions

In conclusion, adhering to traditional northern (high intakes of wheat, other staple and eggs, moderate intakes of dairy products, and a low intake of rice and preserved vegetables) or modern dietary pattern (high intakes of fresh fruit, meat, poultry, fish, dairy products and soybean) was associated with lower risks of CVD and diabetes in the Chinese adults. This study illustrated Chinese dietary patterns that integrated traditional foods like soybean products. Hence, dietary recommendations can incorporate common local foods which would be more viable for preventing cardiometabolic diseases. Future studies could generate a dietary index based on the characteristics of traditional northern and modern dietary patterns to assess the overall diet quality, its trend and potential health benefits in the Chinese population.

## Supplementary Information

**Additional file 1.**

## Data Availability

The access policy and procedures are available at www.ckbiobank.org

## Abbreviations

HR: hazard ratio
CI: confidence interval
CVD: cardiovascular diseases
MCE: major coronary events
HS: haemorrhagic stroke
IS: ischaemic stroke
AMI: acute myocardial infarction
IHD: ischaemic heart disease
CBD: cerebrovascular diseases
DASH: Dietary Approaches to Stop Hypertension
FFQ: food frequency questionnaire
BMI: body mass index
MET: metabolic equivalence task
