# Supplementary material for: Dietary patterns and cardiometabolic diseases in 0.5 million Chinese adults: a 10-year cohort study
Source: Nutr J. 2021 Sep 3;20:74. doi: 10.1186/s12937-021-00730-4 (PMC8418004; doi:10.1186/s12937-021-00730-4)
Supplement: Supplementary file 1 — Additional file 1. [file 12937_2021_730_MOESM1_ESM.docx]

**Supplemental Files**

**Dietary patterns and cardiometabolic diseases in 0.5 million Chinese adults: a 10-year cohort study**

[**Supplemental Table 1 Factor loadings of major dietary patterns using factor analysis** 35](#_Toc34506226)

[**Supplemental Table 2 Daily intake of 12 food groups by the quintiles of dietary pattern scores** 36](#_Toc34506227)

[**Supplemental Table 3 Hazard ratios of secondary endpoints by quintiles of the traditional northern dietary pattern among 477,465 participants** 37](#_Toc34506228)

[**Supplemental Table 4 Hazard ratios of secondary endpoints by quintiles of the modern dietary pattern among 477,465 participants** 39](#_Toc34506229)

[**Supplemental Table 5 Hazard ratios of cardiometabolic diseases by quintiles of two dietary patterns after excluding cases occurring in the first 2 years of follow-up** 41](#_Toc34506230)

[**Supplemental Table 6 Hazard ratios of cardiometabolic diseases by quintiles of the traditional northern dietary patterns after further adjustment** 43](#_Toc34506231)

[**Supplemental Table 7 Hazard ratios of cardiometabolic diseases by quintiles of the modern dietary patterns after further adjustment** 45](#_Toc34506232)

[**Supplemental Table 8 Factor loadings of two dietary patterns after excluding other staples and fresh vegetables.** 47](#_Toc34506233)

[**Supplemental Table 9 Hazard ratios of cardiometabolic diseases by quintiles of two dietary patterns without other staples and fresh vegetables.** 48](#_Toc34506234)

[**Supplemental Table 10 Joint effect of two dietary patterns on cardiometabolic diseases** 50](#_Toc34506235)

[**Supplemental Figure 1 Subgroup analyses of the association between two dietary patterns and cardiovascular disease according to baseline characteristics** 54](#_Toc34506236)

[**Supplemental Figure 2 Subgroup analyses of the association between two dietary patterns and major coronary events according to baseline characteristics** 58](#_Toc34506237)

[**Supplemental Figure 3 Subgroup analyses of the association between two dietary patterns and haemorrhagic stroke according to baseline characteristics** 60](#_Toc34506238)

[**Supplemental Figure 4 Subgroup analyses of the association between two dietary patterns and ischaemic stroke according to baseline characteristics** 62](#_Toc34506239)

[**Supplemental Figure 5 Subgroup analyses of the association between two dietary patterns and diabetes according to baseline characteristics** 64](#_Toc34506240)

[**Members of the China Kadoorie Biobank collaborative group:** 65](#_Toc34506241)

**Supplemental Table 1 Factor loadings of major dietary patterns using factor analysis**

| **Food groups** | **Traditional northern dietary pattern** | **Modern dietary pattern** |
| --- | --- | --- |
| **Rice** | -0.83 | 0.24 |
| **Wheat** | 0.88 | 0.02 |
| **Other staples** | 0.70 | -0.27 |
| **Meat** | -0.13 | 0.69 |
| **Poultry** | -0.19 | 0.62 |
| **Fish** | -0.33 | 0.51 |
| **Eggs** | 0.43 | 0.34 |
| **Fresh vegetables** | 0.14 | 0.20 |
| **Fresh fruit** | 0.08 | 0.72 |
| **Soybean products** | -0.17 | 0.49 |
| **Preserved vegetables** | -0.44 | 0.01 |
| **Dairy products** | 0.34 | 0.51 |
| **Variance explained (%)** | 24.8 | 17.5 |

**Supplemental Table 2 Daily intake of 12 food groups by the quintiles of dietary pattern scores**

| **Food groups (g/d)** | **Traditional northern dietary pattern** | | | | |  | **Modern dietary pattern** | | | | |
| --- | --- | --- | --- | --- | --- | --- | --- | --- | --- | --- | --- |
|  | **Q1** | **Q2** | **Q3** | **Q4** | **Q5** |  | **Q1** | **Q2** | **Q3** | **Q4** | **Q5** |
| Rice | 240.9 | 240.2 | 238.2 | 146.0 | 58.9 |  | 126.1 | 184.5 | 203.9 | 209.4 | 200.3 |
| Wheat | 6.0 | 10.8 | 27.2 | 57.2 | 69.9 |  | 44.3 | 28.0 | 26.4 | 31.3 | 41.0 |
| Other staple | 4.0 | 5.8 | 9.7 | 18.4 | 87.9 |  | 55.2 | 24.1 | 14.4 | 14.3 | 17.7 |
| Meat | 75.0 | 88.9 | 109.3 | 101.4 | 55.8 |  | 26.4 | 52.8 | 83.7 | 114.5 | 153.0 |
| Poultry | 21.7 | 18.3 | 23.1 | 20.4 | 7.2 |  | 1.5 | 5.6 | 13.1 | 25.6 | 44.9 |
| Fish | 23.9 | 18.6 | 21.5 | 15.7 | 5.0 |  | 2.3 | 8.8 | 17.3 | 25.4 | 30.9 |
| Eggs | 24.4 | 33.6 | 49.7 | 55.9 | 72.1 |  | 26.7 | 42.3 | 48.1 | 50.8 | 67.7 |
| Fresh vegetables | 89.8 | 98.2 | 101.9 | 97.5 | 100.1 |  | 93.2 | 96.3 | 96.6 | 99.2 | 102.2 |
| Fresh fruit | 25.5 | 31.3 | 49.9 | 59.1 | 33.0 |  | 10.0 | 19.1 | 30.0 | 50.6 | 89.0 |
| Soybean | 24.8 | 23.7 | 26.0 | 25.5 | 16.4 |  | 7.7 | 21.1 | 26.2 | 28.6 | 32.8 |
| Preserved vegetables | 184.7 | 67.7 | 57.2 | 57.6 | 24.5 |  | 57.5 | 84.3 | 93.8 | 91.7 | 64.5 |
| Dairy products | 2.3 | 4.9 | 15.4 | 39.8 | 44.0 |  | 1.4 | 3.8 | 7.7 | 22.8 | 70.7 |
| Energy intake (kcal/d) | 1486.7 | 1540.2 | 1747.0 | 1517.8 | 1168.3 |  | 938.6 | 1221.8 | 1469.7 | 1735.2 | 2094.8 |

**Supplemental Table 3 Hazard ratios of secondary endpoints by quintiles of the traditional northern dietary pattern among 477,465 participants**

| **Endpoints** | **Traditional northern dietary pattern** | | | | | ***P _trend_*** |
| --- | --- | --- | --- | --- | --- | --- |
|  | **Q1** | **Q2** | **Q3** | **Q4** | **Q5** |  |
| IHD |  |  |  |  |  |  |
| Cases | 5,630 | 7,783 | 8,607 | 12,371 | 10,267 |  |
| Incidence density (1/1,000 PYs) | 6.5 | 6.6 | 6.4 | 6.4 | 6.1 |  |
| Model 1 | 1.00 (Ref.) | 1.03 (0.99, 1.07) | 1.01 (0.97, 1.05) | 1.02 (0.98, 1.07) | 0.99 (0.94, 1.05) | 0.495 |
| Model 2 | 1.00 (Ref.) | 1.01 (0.98, 1.05) | 0.98 (0.94, 1.02) | 1.00 (0.96, 1.05) | 0.98 (0.93, 1.04) | 0.597 |
| Model 3 | 1.00 (Ref.) | 1.02 (0.98, 1.06) | 0.98 (0.94, 1.02) | 1.00 (0.96, 1.05) | 0.99 (0.93, 1.05) | 0.671 |
| AMI |  |  |  |  |  |  |
| Cases | 724 | 1,102 | 1,146 | 1,609 | 1,833 |  |
| Incidence density (1/1,000 PYs) | 0.7 | 0.7 | 0.7 | 0.7 | 0.7 |  |
| Model 1 | 1.00 (Ref.) | 1.01 (0.91, 1.12) | 1.01 (0.91, 1.13) | 1.00 (0.88, 1.13) | 1.02 (0.87, 1.19) | 0.846 |
| Model 2 | 1.00 (Ref.) | 1.00 (0.90, 1.10) | 0.99 (0.89, 1.10) | 0.99 (0.87, 1.12) | 1.03 (0.88, 1.20) | 0.675 |
| Model 3 | 1.00 (Ref.) | 1.01 (0.91, 1.11) | 0.97 (0.88, 1.08) | 0.98 (0.86, 1.11) | 1.04 (0.89, 1.21) | 0.592 |
| CBD |  |  |  |  |  |  |
| Cases | 10,410 | 13,819 | 15,355 | 17,199 | 17,169 |  |
| Incidence density (1/1,000 PYs) | 11.7 | 11.5 | 11.6 | 10.8 | 9.9 |  |
| Model 1 | 1.00 (Ref.) | 1.00 (0.97, 1.03) | 1.01 (0.98, 1.04) | 0.96 (0.93, 1.00) | 0.88 (0.84, 0.93) | <0.001 |
| Model 2 | 1.00 (Ref.) | 0.99 (0.97, 1.02) | 0.99 (0.96, 1.02) | 0.94 (0.91, 0.98) | 0.87 (0.83, 0.92) | <0.001 |
| Model 3 | 1.00 (Ref.) | 1.00 (0.98, 1.03) | 0.98 (0.96, 1.01) | 0.95 (0.92, 0.99) | 0.90 (0.86, 0.94) | <0.001 |
| Total stroke |  |  |  |  |  |  |
| Cases | 7,367 | 8,745 | 9,825 | 12,393 | 12,479 |  |
| Incidence density (1/1,000 PYs) | 8.1 | 7.9 | 7.6 | 6.8 | 6.1 |  |
| Model 1 | 1.00 (Ref.) | 1.01 (0.97, 1.04) | 0.99 (0.95, 1.02) | 0.91 (0.87, 0.95) | 0.81 (0.77, 0.86) | <0.001 |
| Model 2 | 1.00 (Ref.) | 1.00 (0.96, 1.03) | 0.97 (0.93, 1.00) | 0.90 (0.87, 0.94) | 0.82 (0.78, 0.87) | <0.001 |
| Model 3 | 1.00 (Ref.) | 1.01 (0.98, 1.05) | 0.97 (0.93, 1.00) | 0.92 (0.88, 0.96) | 0.86 (0.81, 0.91) | <0.001 |
| Subarachnoid stroke |  |  |  |  |  |  |
| Cases | 155 | 201 | 198 | 191 | 167 |  |
| Incidence density (1/1,000 PYs) | 0.1 | 0.1 | 0.1 | 0.1 | 0.1 |  |
| Model 1 | 1.00 (Ref.) | 1.09 (0.87, 1.38) | 1.10 (0.85, 1.41) | 1.15 (0.84, 1.56) | 0.94 (0.60, 1.45) | 0.993 |
| Model 2 | 1.00 (Ref.) | 1.10 (0.87, 1.38) | 1.09 (0.85, 1.41) | 1.16 (0.85, 1.59) | 0.96 (0.62, 1.50) | 0.891 |
| Model 3 | 1.00 (Ref.) | 1.12 (0.89, 1.41) | 1.11 (0.86, 1.43) | 1.20 (0.88, 1.64) | 1.03 (0.66, 1.60) | 0.696 |
| Pulmonary heart disease |  |  |  |  |  |  |
| Cases | 1,283 | 1,607 | 1,038 | 783 | 640 |  |
| Incidence density (1/1,000 PYs) | 0.3 | 0.3 | 0.3 | 0.2 | 0.2 |  |
| Model 1 | 1.00 (Ref.) | 1.13 (1.05, 1.23) | 1.01 (0.93, 1.11) | 1.00 (0.86, 1.15) | 1.00 (0.82, 1.21) | 0.722 |
| Model 2 | 1.00 (Ref.) | 1.09 (1.01, 1.18) | 1.03 (0.94, 1.13) | 1.06 (0.91, 1.23) | 1.09 (0.89, 1.33) | 0.441 |
| Model 3 | 1.00 (Ref.) | 1.09 (1.01, 1.18) | 1.04 (0.95, 1.14) | 1.06 (0.92, 1.23) | 1.10 (0.90, 1.34) | 0.411 |

Incidence density was adjusted for age at recruitment, sex and survey sites. Hazard ratios (HRs) were estimated using Cox models with stratification on survey sites and age-at-risk (5-year groups). Model 1 was adjusted for sex, age at recruitment, education level. Model 2 was additionally adjusted for smoking, alcohol consumption, physical activity, the average daily energy intake, spicy food, family history of CVD or diabetes, body mass index, and waist circumference. Model 3 was additionally adjusted for prevalent diabetes, antihypertensive drugs use, and systolic blood pressure. Tests for linear trend were conducted by assigning the median value to each quintile and modelling it as a continuous variable in the Cox model.

IHD: ischaemic heart disease. AMI: acute myocardial infarction. CBD: cerebrovascular disease. PY: person year.

* Analyses were performed among 451,846 diabetic participants.

**Supplemental Table 4 Hazard ratios of secondary endpoints by quintiles of the modern dietary pattern among 477,465 participants**

| **Endpoints** | **Modern dietary pattern** | | | | | ***P _trend_*** |
| --- | --- | --- | --- | --- | --- | --- |
|  | **Q1** | **Q2** | **Q3** | **Q4** | **Q5** |  |
| IHD |  |  |  |  |  |  |
| Cases | 9,429 | 8,210 | 8,033 | 8,931 | 10,055 |  |
| Incidence density (1/1,000 PYs) | 6.4 | 6.3 | 6.3 | 6.5 | 6.4 |  |
| Model 1 | 1.00 (Ref.) | 1.00 (0.97, 1.03) | 1.02 (0.99, 1.06) | 1.04 (1.00, 1.08) | 1.03 (0.98, 1.07) | 0.143 |
| Model 2 | 1.00 (Ref.) | 1.00 (0.96, 1.03) | 1.00 (0.96, 1.04) | 1.01 (0.96, 1.05) | 1.01 (0.95, 1.06) | 0.743 |
| Model 3 | 1.00 (Ref.) | 1.00 (0.97, 1.03) | 1.00 (0.97, 1.04) | 1.01 (0.97, 1.06) | 1.02 (0.97, 1.08) | 0.446 |
| AMI |  |  |  |  |  |  |
| Cases | 1,652 | 1,256 | 1,148 | 1,166 | 1,192 |  |
| Incidence density (1/1,000 PYs) | 0.8 | 0.7 | 0.7 | 0.7 | 0.7 |  |
| Model 1 | 1.00 (Ref.) | 0.94 (0.87, 1.02) | 0.94 (0.87, 1.03) | 0.97 (0.88, 1.06) | 0.91 (0.81, 1.01) | 0.128 |
| Model 2 | 1.00 (Ref.) | 0.94 (0.86, 1.01) | 0.91 (0.83, 1.00) | 0.92 (0.82, 1.03) | 0.88 (0.76, 1.01) | 0.107 |
| Model 3 | 1.00 (Ref.) | 0.95 (0.87, 1.03) | 0.92 (0.84, 1.02) | 0.94 (0.83, 1.05) | 0.90 (0.78, 1.05) | 0.217 |
| CBD |  |  |  |  |  |  |
| Cases | 16,941 | 14,698 | 14,587 | 14,671 | 13,055 |  |
| Incidence density (1/1,000 PYs) | 11.2 | 11.1 | 11.4 | 11.5 | 10.4 |  |
| Model 1 | 1.00 (Ref.) | 1.01 (0.98, 1.03) | 1.03 (1.00, 1.06) | 1.03 (1.01, 1.06) | 0.95 (0.92, 0.98) | 0.006 |
| Model 2 | 1.00 (Ref.) | 0.98 (0.96, 1.01) | 0.98 (0.95, 1.00) | 0.95 (0.92, 0.98) | 0.86 (0.82, 0.90) | <0.001 |
| Model 3 | 1.00 (Ref.) | 1.00 (0.97, 1.02) | 0.99 (0.96, 1.02) | 0.97 (0.94, 1.00) | 0.88 (0.84, 0.92) | <0.001 |
| Total stroke |  |  |  |  |  |  |
| Cases | 12,117 | 9,861 | 9,404 | 10,262 | 9,165 |  |
| Incidence density (1/1,000 PYs) | 7.8 | 7.6 | 7.4 | 7.4 | 6.4 |  |
| Model 1 | 1.00 (Ref.) | 0.98 (0.95, 1.01) | 0.96 (0.93, 0.99) | 0.97 (0.94, 1.00) | 0.85 (0.82, 0.89) | <0.001 |
| Model 2 | 1.00 (Ref.) | 0.96 (0.93, 0.99) | 0.91 (0.88, 0.94) | 0.90 (0.86, 0.93) | 0.78 (0.74, 0.83) | <0.001 |
| Model 3 | 1.00 (Ref.) | 0.98 (0.95, 1.00) | 0.93 (0.90, 0.96) | 0.92 (0.88, 0.96) | 0.82 (0.78, 0.86) | <0.001 |
| Subarachnoid stroke |  |  |  |  |  |  |
| Cases | 194 | 177 | 178 | 184 | 179 |  |
| Incidence density (1/1,000 PYs) | 0.1 | 0.1 | 0.1 | 0.1 | 0.1 |  |
| Model 1 | 1.00 (Ref.) | 0.91 (0.73, 1.13) | 0.93 (0.74, 1.17) | 1.01 (0.78, 1.29) | 0.98 (0.73, 1.31) | 0.882 |
| Model 2 | 1.00 (Ref.) | 0.91 (0.73, 1.14) | 0.94 (0.73, 1.22) | 1.02 (0.75, 1.39) | 1.02 (0.69, 1.50) | 0.780 |
| Model 3 | 1.00 (Ref.) | 0.92 (0.74, 1.16) | 0.95 (0.74, 1.24) | 1.05 (0.77, 1.43) | 1.06 (0.71, 1.56) | 0.632 |
| Pulmonary heart disease |  |  |  |  |  |  |
| Cases | 1,985 | 1,489 | 1,012 | 547 | 318 |  |
| Incidence density (1/1,000 PYs) | 0.4 | 0.3 | 0.3 | 0.2 | 0.2 |  |
| Model 1 | 1.00 (Ref.) | 0.84 (0.78, 0.90) | 0.77 (0.71, 0.83) | 0.65 (0.58, 0.72) | 0.63 (0.54, 0.73) | <0.001 |
| Model 2 | 1.00 (Ref.) | 0.89 (0.82, 0.95) | 0.86 (0.78, 0.95) | 0.76 (0.67, 0.88) | 0.79 (0.65, 0.97) | 0.001 |
| Model 3 | 1.00 (Ref.) | 0.89 (0.82, 0.96) | 0.86 (0.78, 0.95) | 0.77 (0.67, 0.88) | 0.80 (0.65, 0.97) | 0.001 |

Incidence density was adjusted for age at recruitment, sex and survey sites. Hazard ratios (HRs) were estimated using Cox models with stratification on survey sites and age-at-risk (5-year groups). Model 1 was adjusted for sex, age at recruitment, education level. Model 2 was additionally adjusted for smoking, alcohol consumption, physical activity, the average daily energy intake, spicy food, family history of CVD or diabetes, body mass index, and waist circumference. Model 3 was additionally adjusted for prevalent diabetes, antihypertensive drugs use, and systolic blood pressure. Tests for linear trend were conducted by assigning the median value to each quintile and modelling it as a continuous variable in the Cox model.

IHD: ischaemic heart disease. AMI: acute myocardial infarction. CBD: cerebrovascular disease. PY: person year.

* Analyses were performed among 451,846 diabetic participants.

**Supplemental Table 5 Hazard ratios of cardiometabolic diseases by quintiles of two dietary patterns after excluding cases occurring in the first 2 years of follow-up**

| **Endpoints** | **Q1** | **Q2** | **Q3** | **Q4** | **Q5** | ***P _trend_*** |
| --- | --- | --- | --- | --- | --- | --- |
| **Traditional northern dietary pattern** | | |  |  |  |  |
| CVD | 1.00 (Ref.) | 0.99 (0.97, 1.01) | 0.98 (0.96, 1.00) | 0.95 (0.92, 0.98) | 0.93 (0.89, 0.96) | <0.001 |
| IHD | 1.00 (Ref.) | 1.04 (1.00, 1.08) | 1.01 (0.96, 1.05) | 1.02 (0.97, 1.07) | 0.99 (0.93, 1.06) | 0.478 |
| MCE | 1.00 (Ref.) | 1.00 (0.91, 1.09) | 0.96 (0.88, 1.06) | 0.89 (0.80, 1.00) | 0.90 (0.79, 1.03) | 0.091 |
| AMI | 1.00 (Ref.) | 0.99 (0.89, 1.10) | 0.98 (0.88, 1.09) | 0.96 (0.84, 1.10) | 1.00 (0.85, 1.18) | 0.931 |
| CBD | 1.00 (Ref.) | 1.01 (0.98, 1.04) | 0.99 (0.96, 1.02) | 0.95 (0.91, 0.99) | 0.89 (0.85, 0.94) | <0.001 |
| Total stroke | 1.00 (Ref.) | 1.02 (0.98, 1.06) | 0.97 (0.93, 1.01) | 0.90 (0.86, 0.94) | 0.84 (0.79, 0.89) | <0.001 |
| Subarachnoid stroke | 1.00 (Ref.) | 1.20 (0.94, 1.55) | 1.22 (0.93, 1.61) | 1.22 (0.87, 1.70) | 1.01 (0.63, 1.61) | 0.894 |
| HS | 1.00 (Ref.) | 1.00 (0.93, 1.08) | 0.89 (0.82, 0.96) | 0.87 (0.78, 0.97) | 0.75 (0.65, 0.86) | <0.001 |
| IS | 1.00 (Ref.) | 1.02 (0.98, 1.06) | 0.98 (0.94, 1.02) | 0.90 (0.85, 0.94) | 0.85 (0.80, 0.90) | <0.001 |
| Pulmonary heart disease | 1.00 (Ref.) | 1.17 (1.08, 1.28) | 1.10 (0.99, 1.21) | 1.17 (1.00, 1.37) | 1.23 (0.99, 1.51) | 0.061 |
| Diabetes* | 1.00 (Ref.) | 0.92 (0.88, 0.97) | 0.93 (0.88, 0.98) | 0.81 (0.75, 0.87) | 0.84 (0.75, 0.94) | <0.001 |
| **Modern dietary pattern** |  |  |  |  |  |  |
| CVD | 1.00 (Ref.) | 0.98 (0.96, 0.99) | 0.97 (0.95, 0.99) | 0.97 (0.94, 0.99) | 0.91 (0.88, 0.95) | <0.001 |
| IHD | 1.00 (Ref.) | 1.00 (0.97, 1.03) | 1.01 (0.97, 1.05) | 1.03 (0.98, 1.08) | 1.03 (0.97, 1.09) | 0.270 |
| MCE | 1.00 (Ref.) | 0.95 (0.88, 1.02) | 0.93 (0.86, 1.01) | 0.92 (0.83, 1.02) | 0.85 (0.75, 0.97) | 0.019 |
| AMI | 1.00 (Ref.) | 0.96 (0.88, 1.05) | 0.92 (0.83, 1.02) | 0.94 (0.83, 1.06) | 0.86 (0.73, 1.00) | 0.062 |
| CBD | 1.00 (Ref.) | 0.99 (0.97, 1.02) | 0.99 (0.96, 1.02) | 0.97 (0.93, 1.00) | 0.87 (0.84, 0.91) | <0.001 |
| Total stroke | 1.00 (Ref.) | 0.98 (0.95, 1.01) | 0.93 (0.90, 0.96) | 0.91 (0.87, 0.95) | 0.80 (0.76, 0.85) | <0.001 |
| Subarachnoid stroke | 1.00 (Ref.) | 0.99 (0.78, 1.25) | 1.00 (0.76, 1.32) | 1.09 (0.78, 1.51) | 1.13 (0.74, 1.70) | 0.508 |
| HS | 1.00 (Ref.) | 0.97 (0.91, 1.03) | 0.90 (0.83, 0.98) | 0.81 (0.73, 0.90) | 0.68 (0.59, 0.78) | <0.001 |
| IS | 1.00 (Ref.) | 0.99 (0.96, 1.02) | 0.95 (0.91, 0.99) | 0.94 (0.90, 0.99) | 0.83 (0.78, 0.88) | <0.001 |
| Pulmonary heart disease | 1.00 (Ref.) | 0.91 (0.84, 0.99) | 0.89 (0.80, 0.98) | 0.81 (0.70, 0.93) | 0.83 (0.67, 1.02) | 0.013 |
| Diabetes* | 1.00 (Ref.) | 0.92 (0.87, 0.98) | 0.92 (0.86, 0.98) | 0.88 (0.82, 0.95) | 0.88 (0.80, 0.97) | 0.016 |

Incidence density was adjusted for age at recruitment, sex and survey sites. Hazard ratios (HRs) were estimated using Cox models with stratification on survey sites and age-at-risk (5-year groups), and adjustment for sex, age at recruitment, education level, smoking, alcohol consumption, physical activity, the average daily energy intake, spicy food, family history of CVD or diabetes, body mass index, waist circumference, prevalent diabetes, antihypertensive drugs use, and systolic blood pressure. Tests for linear trend were conducted by assigning the median value to each quintile and modelling it as a continuous variable in the Cox model.

CVD: cardiovascular disease. MCE: major coronary events. IHD: ischaemic heart disease. AMI: acute myocardial infarction. CBD: cerebrovascular disease. HS: haemorrhagic stroke. IS: ischaemic stroke.

* Analyses were performed among diabetic participants.

**Supplemental Table 6 Hazard ratios of cardiometabolic diseases by quintiles of the traditional northern dietary patterns after further adjustment**

| **Endpoints** | **Q1** | | **Q2** | | **Q3** | | **Q4** | | **Q5** | | ***P _trend_*** | |  |
| --- | --- | --- | --- | --- | --- | --- | --- | --- | --- | --- | --- | --- | --- |
| CVD |  | |  | |  | |  | |  | |  | |  |
| Model 4 | 1.00 (Ref.) | | 0.97 (0.96, 0.99) | | 0.96 (0.94, 0.98) | | 0.94 (0.92, 0.97) | | 0.92 (0.89, 0.95) | | <0.001 | |  |
| Model 5 | 1.00 (Ref.) | | 0.97 (0.96, 0.99) | | 0.96 (0.94, 0.98) | | 0.94 (0.92, 0.97) | | 0.92 (0.89, 0.95) | | <0.001 | |  |
| IHD |  | |  | |  | |  | |  | |  | |  |
| Model 4 | 1.00 (Ref.) | | 1.02 (0.98, 1.06) | | 0.98 (0.94, 1.02) | | 1.00 (0.95, 1.05) | | 0.98 (0.93, 1.04) | | 0.506 | |  |
| Model 5 | 1.00 (Ref.) | | 1.02 (0.98, 1.06) | | 0.98 (0.94, 1.01) | | 1.00 (0.95, 1.05) | | 0.98 (0.93, 1.04) | | 0.505 | |  |
| MCE | |  | |  | |  | |  | |  | |  | |
| Model 4 | | 1.00 (Ref.) | | 1.01 (0.93, 1.10) | | 0.95 (0.87, 1.04) | | 0.89 (0.80, 1.00) | | 0.91 (0.80, 1.04) | | 0.086 | |
| Model 5 | | 1.00 (Ref.) | | 1.01 (0.93, 1.10) | | 0.95 (0.87, 1.04) | | 0.89 (0.80, 1.00) | | 0.91 (0.80, 1.04) | | 0.086 | |
| AMI |  | |  | |  | |  | |  | |  | |  |
| Model 4 | 1.00 (Ref.) | | 1.00 (0.91, 1.11) | | 0.97 (0.87, 1.08) | | 0.97 (0.85, 1.11) | | 1.03 (0.88, 1.20) | | 0.678 | |  |
| Model 5 | 1.00 (Ref.) | | 1.01 (0.91, 1.11) | | 0.97 (0.87, 1.08) | | 0.97 (0.86, 1.11) | | 1.03 (0.88, 1.20) | | 0.679 | |  |
| CBD |  | |  | |  | |  | |  | |  | |  |
| Model 4 | 1.00 (Ref.) | | 1.00 (0.98, 1.03) | | 0.98 (0.95, 1.01) | | 0.95 (0.92, 0.98) | | 0.89 (0.85, 0.94) | | <0.001 | |  |
| Model 5 | 1.00 (Ref.) | | 1.00 (0.98, 1.03) | | 0.98 (0.95, 1.01) | | 0.95 (0.92, 0.98) | | 0.89 (0.85, 0.94) | | <0.001 | |  |
| Total stroke |  | |  | |  | |  | |  | |  | |  |
| Model 4 | 1.00 (Ref.) | | 1.01 (0.98, 1.05) | | 0.96 (0.93, 1.00) | | 0.91 (0.88, 0.95) | | 0.85 (0.81, 0.90) | | <0.001 | |  |
| Model 5 | 1.00 (Ref.) | | 1.01 (0.98, 1.05) | | 0.96 (0.93, 1.00) | | 0.91 (0.88, 0.95) | | 0.85 (0.81, 0.90) | | <0.001 | |  |
| Subarachnoid stroke |  | |  | |  | |  | |  | |  | |  |
| Model 4 | 1.00 (Ref.) | | 1.12 (0.89, 1.41) | | 1.11 (0.86, 1.43) | | 1.20 (0.88, 1.63) | | 1.02 (0.65, 1.58) | | 0.724 | |  |
| Model 5 | 1.00 (Ref.) | | 1.12 (0.89, 1.41) | | 1.11 (0.86, 1.43) | | 1.20 (0.88, 1.63) | | 1.02 (0.65, 1.58) | | 0.723 | |  |
| HS |  | |  | |  | |  | |  | |  | |  |
| Model 4 | 1.00 (Ref.) | | 1.00 (0.93, 1.07) | | 0.89 (0.82, 0.96) | | 0.88 (0.80, 0.98) | | 0.77 (0.68, 0.89) | | <0.001 | |  |
| Model 5 | 1.00 (Ref.) | | 1.00 (0.93, 1.07) | | 0.89 (0.82, 0.96) | | 0.88 (0.80, 0.98) | | 0.77 (0.68, 0.89) | | <0.001 | |  |
| IS |  | |  | |  | |  | |  | |  | |  |
| Model 4 | 1.00 (Ref.) | | 1.00 (0.97, 1.04) | | 0.97 (0.93, 1.01) | | 0.91 (0.87, 0.95) | | 0.86 (0.81, 0.91) | | <0.001 | |  |
| Model 5 | 1.00 (Ref.) | | 1.00 (0.97, 1.04) | | 0.97 (0.93, 1.01) | | 0.91 (0.87, 0.95) | | 0.86 (0.81, 0.91) | | <0.001 | |  |
| Pulmonary heart disease |  | |  | |  | |  | |  | |  | |  |
| Model 4 | 1.00 (Ref.) | | 1.09 (1.01, 1.18) | | 1.04 (0.95, 1.14) | | 1.06 (0.92, 1.23) | | 1.10 (0.90, 1.34) | | 0.406 | |  |
| Model 5 | 1.00 (Ref.) | | 1.09 (1.01, 1.18) | | 1.04 (0.95, 1.14) | | 1.06 (0.92, 1.23) | | 1.10 (0.90, 1.34) | | 0.405 | |  |
| Diabetes* |  | |  | |  | |  | |  | |  | |  |
| Model 4 | 1.00 (Ref.) | | 0.91 (0.87, 0.96) | | 0.93 (0.88, 0.98) | | 0.81 (0.75, 0.87) | | 0.84 (0.76, 0.94) | | <0.001 | |  |
| Model 5 | 1.00 (Ref.) | | 0.91 (0.87, 0.96) | | 0.93 (0.88, 0.98) | | 0.81 (0.75, 0.87) | | 0.84 (0.76, 0.94) | | <0.001 | |  |

Incidence density was adjusted for age at recruitment, sex and survey sites. Hazard ratios (HRs) were estimated using Cox models with stratification on survey sites and age-at-risk (5-year groups), and adjustment for sex, age at recruitment, education level, smoking, alcohol consumption, physical activity, the average daily energy intake, spicy food, family history of CVD or diabetes, body mass index, waist circumference, prevalent diabetes, antihypertensive drugs use, and systolic blood pressure. Model 4 was additionally adjusted for statin use based on the final model, and model 5 was additionally adjusted for aspirin use Tests for linear trend were conducted by assigning the median value to each quintile and modelling it as a continuous variable in the Cox model.

CVD: cardiovascular disease. MCE: major coronary events. IHD: ischaemic heart disease. AMI: acute myocardial infarction. CBD: cerebrovascular disease. HS: haemorrhagic stroke. IS: ischaemic stroke.

* Analyses were performed in diabetic participants.

**Supplemental Table 7 Hazard ratios of cardiometabolic diseases by quintiles of the modern dietary patterns after further adjustment**

| **Endpoints** | **Q1** | | **Q2** | | **Q3** | | **Q4** | | **Q5** | | ***P _trend_*** | |  |
| --- | --- | --- | --- | --- | --- | --- | --- | --- | --- | --- | --- | --- | --- |
| CVD |  | |  | |  | |  | |  | |  | |  |
| Model 4 | 1.00 (Ref.) | | 0.98 (0.96, 1.00) | | 0.95 (0.93, 0.97) | | 0.93 (0.90, 0.95) | | 0.88 (0.86, 0.91) | | <0.001 | |  |
| Model 5 | 1.00 (Ref.) | | 0.98 (0.96, 1.00) | | 0.95 (0.93, 0.97) | | 0.93 (0.90, 0.95) | | 0.88 (0.86, 0.91) | | <0.001 | |  |
| IHD |  | |  | |  | |  | |  | |  | |  |
| Model 4 | 1.00 (Ref.) | | 1.00 (0.96, 1.03) | | 1.00 (0.96, 1.04) | | 1.01 (0.96, 1.05) | | 1.01 (0.96, 1.07) | | 0.605 | |  |
| Model 5 | 1.00 (Ref.) | | 1.00 (0.96, 1.03) | | 1.00 (0.96, 1.04) | | 1.01 (0.96, 1.05) | | 1.01 (0.96, 1.07) | | 0.608 | |  |
| MCE | |  | |  | |  | |  | |  | |  | |
| Model 4 | | 1.00 (Ref.) | | 0.94 (0.87, 1.00) | | 0.93 (0.85, 1.00) | | 0.91 (0.82, 1.00) | | 0.87 (0.77, 0.98) | | 0.036 | |
| Model 5 | | 1.00 (Ref.) | | 0.94 (0.87, 1.00) | | 0.93 (0.85, 1.00) | | 0.91 (0.82, 1.00) | | 0.87 (0.77, 0.98) | | 0.035 | |
| AMI |  | |  | |  | |  | |  | |  | |  |
| Model 4 | 1.00 (Ref.) | | 0.94 (0.87, 1.02) | | 0.92 (0.84, 1.01) | | 0.93 (0.83, 1.04) | | 0.90 (0.77, 1.04) | | 0.183 | |  |
| Model 5 | 1.00 (Ref.) | | 0.94 (0.87, 1.02) | | 0.92 (0.84, 1.01) | | 0.93 (0.83, 1.04) | | 0.90 (0.77, 1.04) | | 0.178 | |  |
| CBD |  | |  | |  | |  | |  | |  | |  |
| Model 4 | 1.00 (Ref.) | | 0.99 (0.97, 1.02) | | 0.99 (0.96, 1.01) | | 0.96 (0.93, 1.00) | | 0.88 (0.84, 0.92) | | <0.001 | |  |
| Model 5 | 1.00 (Ref.) | | 0.99 (0.97, 1.02) | | 0.99 (0.96, 1.01) | | 0.96 (0.93, 1.00) | | 0.88 (0.84, 0.92) | | <0.001 | |  |
| Total stroke |  | |  | |  | |  | |  | |  | |  |
| Model 4 | 1.00 (Ref.) | | 0.97 (0.94, 1.00) | | 0.93 (0.90, 0.96) | | 0.92 (0.88, 0.96) | | 0.81 (0.77, 0.86) | | <0.001 | |  |
| Model 5 | 1.00 (Ref.) | | 0.97 (0.95, 1.00) | | 0.93 (0.90, 0.96) | | 0.92 (0.88, 0.96) | | 0.81 (0.77, 0.86) | | <0.001 | |  |
| Subarachnoid stroke |  | |  | |  | |  | |  | |  | |  |
| Model 4 | 1.00 (Ref.) | | 0.92 (0.73, 1.15) | | 0.95 (0.73, 1.23) | | 1.04 (0.77, 1.42) | | 1.04 (0.70, 1.55) | | 0.672 | |  |
| Model 5 | 1.00 (Ref.) | | 0.92 (0.73, 1.15) | | 0.95 (0.73, 1.23) | | 1.04 (0.77, 1.42) | | 1.04 (0.70, 1.54) | | 0.672 | |  |
| HS |  | |  | |  | |  | |  | |  | |  |
| Model 4 | 1.00 (Ref.) | | 0.96 (0.90, 1.02) | | 0.90 (0.84, 0.97) | | 0.82 (0.75, 0.91) | | 0.67 (0.59, 0.77) | | <0.001 | |  |
| Model 5 | 1.00 (Ref.) | | 0.96 (0.90, 1.02) | | 0.90 (0.84, 0.97) | | 0.82 (0.75, 0.91) | | 0.67 (0.59, 0.77) | | <0.001 | |  |
| IS |  | |  | |  | |  | |  | |  | |  |
| Model 4 | 1.00 (Ref.) | | 0.99 (0.95, 1.02) | | 0.95 (0.91, 0.99) | | 0.95 (0.91, 0.99) | | 0.84 (0.80, 0.89) | | <0.001 | |  |
| Model 5 | 1.00 (Ref.) | | 0.99 (0.95, 1.02) | | 0.95 (0.91, 0.99) | | 0.95 (0.91, 0.99) | | 0.84 (0.80, 0.89) | | <0.001 | |  |
| Pulmonary heart disease |  | |  | |  | |  | |  | |  | |  |
| Model 4 | 1.00 (Ref.) | | 0.89 (0.82, 0.96) | | 0.86 (0.78, 0.95) | | 0.77 (0.67, 0.88) | | 0.80 (0.65, 0.98) | | 0.001 | |  |
| Model 5 | 1.00 (Ref.) | | 0.89 (0.82, 0.96) | | 0.86 (0.78, 0.95) | | 0.77 (0.67, 0.88) | | 0.80 (0.65, 0.98) | | 0.001 | |  |
| Diabetes* |  | |  | |  | |  | |  | |  | |  |
| Model 4 | 1.00 (Ref.) | | 0.92 (0.88, 0.97) | | 0.92 (0.87, 0.98) | | 0.88 (0.82, 0.94) | | 0.88 (0.80, 0.96) | | 0.011 | |  |
| Model 5 | 1.00 (Ref.) | | 0.92 (0.88, 0.97) | | 0.92 (0.87, 0.98) | | 0.88 (0.82, 0.94) | | 0.88 (0.80, 0.96) | | 0.011 | |  |

Incidence density was adjusted for age at recruitment, sex and survey sites. Hazard ratios (HRs) were estimated using Cox models with stratification on survey sites and age-at-risk (5-year groups), and adjustment for sex, age at recruitment, education level, smoking, alcohol consumption, physical activity, the average daily energy intake, spicy food, family history of CVD or diabetes, body mass index, waist circumference, prevalent diabetes, antihypertensive drugs use, and systolic blood pressure. Model 4 was additionally adjusted for statin use based on the final model, and model 5 was additionally adjusted for aspirin use Tests for linear trend were conducted by assigning the median value to each quintile and modelling it as a continuous variable in the Cox model.

CVD: cardiovascular disease. MCE: major coronary events. IHD: ischaemic heart disease. AMI: acute myocardial infarction. CBD: cerebrovascular disease. HS: haemorrhagic stroke. IS: ischaemic stroke.

* Analyses were performed in diabetic participants.

**Supplemental Table 8 Factor loadings of two dietary patterns after excluding other staples and fresh vegetables.**

| **Food groups** | **Traditional northern dietary pattern** | **Modern dietary pattern** |
| --- | --- | --- |
| **Rice** | -0.79 | 0.38 |
| **Wheat** | 0.86 | -0.13 |
| **Meat** | -0.004 | 0.68 |
| **Poultry** | -0.12 | 0.66 |
| **Fish** | -0.26 | 0.57 |
| **Eggs** | 0.50 | 0.26 |
| **Fresh fruit** | 0.21 | 0.70 |
| **Soybean products** | -0.09 | 0.54 |
| **Preserved vegetables** | -0.44 | 0.09 |
| **Dairy products** | 0.46 | 0.44 |
| **Variance explained (%)** | 20.0 | 25.8 |

**Supplemental Table 9 Hazard ratios of cardiometabolic diseases by quintiles of two dietary patterns without other staples and fresh vegetables.**

| **Endpoints** | **Q1** | **Q2** | **Q3** | **Q4** | **Q5** | ***P_trend_*** |
| --- | --- | --- | --- | --- | --- | --- |
| **Traditional northern dietary pattern** |  |  |  |  |  |  |
| CVD | 1.00 (Ref.) | 0.99 (0.98, 1.01) | 0.98 (0.96, 1.01) | 0.95 (0.92, 0.97) | 0.92 (0.89, 0.95) | <0.001 |
| IHD | 1.00 (Ref.) | 1.02 (0.99, 1.06) | 1.02 (0.98, 1.07) | 1.03 (0.99, 1.08) | 1.05 (0.99, 1.11) | 0.121 |
| MCE | 1.00 (Ref.) | 1.00 (0.93, 1.07) | 0.95 (0.87, 1.04) | 0.95 (0.85, 1.06) | 0.93 (0.81, 1.06) | 0.239 |
| AMI | 1.00 (Ref.) | 0.99 (0.91, 1.08) | 0.94 (0.85, 1.05) | 0.95 (0.84, 1.07) | 0.96 (0.82, 1.13) | 0.608 |
| CBD | 1.00 (Ref.) | 0.99 (0.97, 1.02) | 1.00 (0.97, 1.03) | 0.97 (0.94, 1.01) | 0.90 (0.86, 0.94) | <0.001 |
| Total stroke | 1.00 (Ref.) | 0.96 (0.93, 0.99) | 0.93 (0.90, 0.97) | 0.91 (0.87, 0.95) | 0.83 (0.79, 0.88) | <0.001 |
| Subarachnoid stroke | 1.00 (Ref.) | 1.13 (0.88, 1.43) | 0.99 (0.74, 1.33) | 1.01 (0.72, 1.42) | 1.08 (0.71, 1.64) | 0.898 |
| HS | 1.00 (Ref.) | 0.90 (0.84, 0.96) | 0.88 (0.81, 0.96) | 0.78 (0.70, 0.86) | 0.67 (0.59, 0.77) | <0.001 |
| IS | 1.00 (Ref.) | 0.98 (0.95, 1.01) | 0.96 (0.92, 1.00) | 0.95 (0.91, 1.00) | 0.86 (0.81, 0.92) | <0.001 |
| Pulmonary heart disease | 1.00 (Ref.) | 0.89 (0.82, 0.96) | 0.82 (0.74, 0.92) | 0.69 (0.60, 0.80) | 0.67 (0.55, 0.83) | <0.001 |
| Diabetes* | 1.00 (Ref.) | 0.93 (0.88, 0.99) | 0.93 (0.87, 1.00) | 0.89 (0.82, 0.96) | 0.88 (0.80, 0.96) | 0.011 |
| **Modern dietary pattern** |  |  |  |  |  |  |
| CVD | 1.00 (Ref.) | 0.97 (0.95, 0.99) | 0.94 (0.92, 0.96) | 0.93 (0.90, 0.95) | 0.93 (0.90, 0.96) | <0.001 |
| IHD | 1.00 (Ref.) | 0.99 (0.96, 1.03) | 0.96 (0.93, 1.00) | 0.97 (0.92, 1.01) | 0.99 (0.94, 1.04) | 0.726 |
| MCE | 1.00 (Ref.) | 1.06 (0.97, 1.15) | 0.96 (0.88, 1.05) | 0.91 (0.81, 1.01) | 0.89 (0.79, 1.00) | 0.007 |
| AMI | 1.00 (Ref.) | 1.03 (0.93, 1.13) | 0.95 (0.86, 1.05) | 0.96 (0.85, 1.09) | 0.93 (0.81, 1.08) | 0.240 |
| CBD | 1.00 (Ref.) | 1.00 (0.97, 1.02) | 0.98 (0.95, 1.01) | 0.95 (0.92, 0.99) | 0.95 (0.91, 0.98) | 0.002 |
| Total stroke | 1.00 (Ref.) | 1.00 (0.97, 1.04) | 0.96 (0.92, 0.99) | 0.91 (0.87, 0.95) | 0.90 (0.86, 0.94) | <0.001 |
| Subarachnoid stroke | 1.00 (Ref.) | 1.20 (0.96, 1.50) | 1.08 (0.84, 1.40) | 1.31 (0.97, 1.78) | 1.19 (0.82, 1.71) | 0.338 |
| HS | 1.00 (Ref.) | 0.97 (0.90, 1.04) | 0.88 (0.81, 0.95) | 0.84 (0.76, 0.93) | 0.75 (0.66, 0.84) | <0.001 |
| IS | 1.00 (Ref.) | 1.00 (0.97, 1.04) | 0.97 (0.93, 1.01) | 0.91 (0.87, 0.96) | 0.91 (0.87, 0.96) | <0.001 |
| Pulmonary heart disease | 1.00 (Ref.) | 1.04 (0.97, 1.12) | 0.99 (0.91, 1.09) | 0.94 (0.81, 1.09) | 1.08 (0.90, 1.30) | 0.808 |
| Diabetes* | 1.00 (Ref.) | 0.92 (0.88, 0.96) | 0.89 (0.85, 0.94) | 0.82 (0.77, 0.88) | 0.84 (0.77, 0.92) | <0.001 |

Hazard ratios (HRs) were estimated using Cox models with stratification on survey sites and age-at-risk (5-year groups), and adjustment for sex, age at recruitment, education level, smoking, alcohol consumption, physical activity, the average daily energy intake, spicy food, family history of CVD or diabetes, body mass index, waist circumference, prevalent diabetes, antihypertensive drugs use, and systolic blood pressure. Tests for linear trend were conducted by assigning the median value to each quintile and modelling it as a continuous variable in the Cox model.

CVD: cardiovascular disease. MCE: major coronary events. IHD: ischaemic heart disease. AMI: acute myocardial infarction. CBD: cerebrovascular disease. HS: haemorrhagic stroke. IS: ischaemic stroke.

* Analyses were performed among diabetic participants.

**Supplemental Table 10 Joint effect of two dietary patterns on cardiometabolic diseases**

| **Traditional northern dietary pattern** | **Modern dietary pattern** | | | | | ***P _interaction_*** |
| --- | --- | --- | --- | --- | --- | --- |
|  | **Q1** | **Q2** | **Q3** | **Q4** | **Q5** |  |
| CVD |  |  |  |  |  |  |
| Q1 | 1.00 (Ref.) | 0.97 (0.94, 1.01) | 0.92 (0.89, 0.96) | 0.90 (0.86, 0.94) | 0.86 (0.79, 0.93) | <0.001 |
| Q2 | 0.99 (0.95, 1.03) | 0.94 (0.90, 0.97) | 0.92 (0.89, 0.96) | 0.87 (0.84, 0.91) | 0.88 (0.83, 0.93) |  |
| Q3 | 0.93 (0.88, 0.98) | 0.95 (0.91, 0.99) | 0.92 (0.89, 0.96) | 0.88 (0.85, 0.92) | 0.84 (0.80, 0.88) |  |
| Q4 | 0.92 (0.87, 0.97) | 0.89 (0.84, 0.93) | 0.88 (0.84, 0.92) | 0.90 (0.87, 0.94) | 0.85 (0.82, 0.89) |  |
| Q5 | 0.86 (0.82, 0.92) | 0.88 (0.83, 0.93) | 0.90 (0.84, 0.95) | 0.90 (0.85, 0.95) | 0.83 (0.79, 0.87) |  |
| MCE |  |  |  |  |  |  |
| Q1 | 1.00 (Ref.) | 0.83 (0.70, 0.99) | 0.86 (0.71, 1.03) | 0.81 (0.65, 1.01) | 1.00 (0.70, 1.40) | 0.168 |
| Q2 | 0.97 (0.83, 1.14) | 0.85 (0.73, 1.00) | 0.89 (0.75, 1.05) | 0.91 (0.75, 1.09) | 0.84 (0.66, 1.07) |  |
| Q3 | 0.80 (0.64, 0.99) | 0.90 (0.76, 1.07) | 0.84 (0.71, 0.99) | 0.82 (0.69, 0.97) | 0.80 (0.66, 0.98) |  |
| Q4 | 0.86 (0.70, 1.05) | 0.83 (0.68, 1.01) | 0.78 (0.65, 0.94) | 0.77 (0.65, 0.92) | 0.76 (0.63, 0.92) |  |
| Q5 | 0.92 (0.74, 1.14) | 0.87 (0.70, 1.08) | 0.87 (0.70, 1.09) | 0.84 (0.69, 1.04) | 0.75 (0.62, 0.92) |  |
| IHD |  |  |  |  |  |  |
| Q1 | 1.00 (Ref.) | 0.99 (0.92, 1.06) | 0.96 (0.89, 1.04) | 1.03 (0.94, 1.13) | 0.99 (0.85, 1.16) | 0.818 |
| Q2 | 1.04 (0.97, 1.12) | 0.99 (0.92, 1.06) | 1.03 (0.95, 1.10) | 1.00 (0.92, 1.08) | 1.01 (0.91, 1.12) |  |
| Q3 | 0.98 (0.89, 1.08) | 0.98 (0.90, 1.05) | 0.98 (0.91, 1.05) | 0.97 (0.90, 1.05) | 0.96 (0.89, 1.05) |  |
| Q4 | 1.00 (0.91, 1.10) | 0.95 (0.87, 1.04) | 0.97 (0.90, 1.05) | 0.99 (0.92, 1.06) | 1.02 (0.94, 1.10) |  |
| Q5 | 0.90 (0.81, 1.00) | 0.96 (0.87, 1.06) | 1.00 (0.90, 1.10) | 1.02 (0.93, 1.12) | 0.98 (0.90, 1.07) |  |
| AMI |  |  |  |  |  |  |
| Q1 | 1.00 (Ref.) | 0.82 (0.67, 1.01) | 0.86 (0.70, 1.07) | 0.89 (0.70, 1.13) | 0.91 (0.60, 1.33) | 0.664 |
| Q2 | 0.96 (0.79, 1.16) | 0.84 (0.70, 1.02) | 0.88 (0.72, 1.07) | 0.97 (0.78, 1.20) | 0.81 (0.61, 1.08) |  |
| Q3 | 0.87 (0.67, 1.12) | 0.97 (0.79, 1.18) | 0.84 (0.69, 1.02) | 0.82 (0.67, 1.01) | 0.82 (0.65, 1.04) |  |
| Q4 | 0.97 (0.77, 1.23) | 0.95 (0.75, 1.20) | 0.80 (0.64, 0.99) | 0.85 (0.69, 1.05) | 0.85 (0.68, 1.06) |  |
| Q5 | 1.07 (0.83, 1.37) | 1.00 (0.78, 1.29) | 1.04 (0.81, 1.34) | 0.95 (0.74, 1.21) | 0.87 (0.68, 1.10) |  |
| CBD |  |  |  |  |  |  |
| Q1 | 1.00 (Ref.) | 0.99 (0.93, 1.04) | 0.96 (0.91, 1.02) | 0.95 (0.89, 1.01) | 0.87 (0.77, 0.98) | 0.167 |
| Q2 | 0.99 (0.94, 1.05) | 0.96 (0.91, 1.02) | 0.99 (0.93, 1.04) | 0.97 (0.92, 1.04) | 0.94 (0.87, 1.02) |  |
| Q3 | 0.97 (0.90, 1.05) | 1.01 (0.95, 1.07) | 0.98 (0.93, 1.04) | 0.94 (0.89, 1.00) | 0.84 (0.79, 0.90) |  |
| Q4 | 0.94 (0.87, 1.02) | 0.94 (0.88, 1.01) | 0.93 (0.88, 0.99) | 0.94 (0.89, 1.00) | 0.86 (0.81, 0.92) |  |
| Q5 | 0.87 (0.81, 0.94) | 0.88 (0.81, 0.95) | 0.89 (0.82, 0.96) | 0.90 (0.83, 0.97) | 0.84 (0.78, 0.90) |  |
| Total stroke |  |  |  |  |  |  |
| Q1 | 1.00 (Ref.) | 1.00 (0.93, 1.06) | 0.96 (0.89, 1.03) | 0.95 (0.87, 1.02) | 0.86 (0.75, 0.98) | 0.327 |
| Q2 | 1.03 (0.96, 1.10) | 0.99 (0.93, 1.06) | 0.97 (0.90, 1.04) | 0.95 (0.88, 1.02) | 0.94 (0.86, 1.04) |  |
| Q3 | 0.95 (0.86, 1.04) | 1.01 (0.94, 1.09) | 0.94 (0.88, 1.00) | 0.94 (0.87, 1.00) | 0.81 (0.75, 0.88) |  |
| Q4 | 0.96 (0.88, 1.04) | 0.93 (0.86, 1.01) | 0.91 (0.84, 0.98) | 0.91 (0.85, 0.98) | 0.80 (0.74, 0.86) |  |
| Q5 | 0.92 (0.84, 1.01) | 0.89 (0.81, 0.97) | 0.84 (0.76, 0.93) | 0.84 (0.77, 0.92) | 0.79 (0.73, 0.86) |  |
| HS |  |  |  |  |  |  |
| Q1 | 1.00 (Ref.) | 0.98 (0.86, 1.11) | 0.95 (0.82, 1.10) | 0.76 (0.63, 0.91) | 0.88 (0.64, 1.19) | 0.012 |
| Q2 | 1.02 (0.90, 1.16) | 0.91 (0.80, 1.03) | 0.95 (0.83, 1.09) | 0.91 (0.77, 1.07) | 0.83 (0.66, 1.04) |  |
| Q3 | 0.81 (0.67, 0.98) | 0.94 (0.82, 1.08) | 0.83 (0.72, 0.95) | 0.81 (0.69, 0.94) | 0.61 (0.51, 0.74) |  |
| Q4 | 0.94 (0.78, 1.12) | 0.86 (0.72, 1.03) | 0.92 (0.78, 1.08) | 0.85 (0.72, 1.00) | 0.63 (0.53, 0.76) |  |
| Q5 | 0.88 (0.72, 1.08) | 0.91 (0.75, 1.11) | 0.70 (0.57, 0.87) | 0.66 (0.53, 0.82) | 0.65 (0.52, 0.80) |  |
| IS |  |  |  |  |  |  |
| Q1 | 1.00 (Ref.) | 1.01 (0.94, 1.09) | 0.98 (0.90, 1.05) | 1.00 (0.91, 1.09) | 0.89 (0.77, 1.03) | 0.261 |
| Q2 | 1.00 (0.93, 1.09) | 1.02 (0.95, 1.10) | 0.98 (0.91, 1.06) | 0.97 (0.89, 1.05) | 0.98 (0.89, 1.09) |  |
| Q3 | 0.98 (0.88, 1.09) | 1.04 (0.96, 1.13) | 0.97 (0.90, 1.05) | 0.97 (0.90, 1.05) | 0.85 (0.78, 0.92) |  |
| Q4 | 0.93 (0.84, 1.02) | 0.95 (0.86, 1.04) | 0.91 (0.84, 0.99) | 0.94 (0.87, 1.02) | 0.83 (0.76, 0.90) |  |
| Q5 | 0.93 (0.84, 1.03) | 0.88 (0.79, 0.97) | 0.88 (0.79, 0.97) | 0.87 (0.79, 0.97) | 0.81 (0.74, 0.89) |  |
| Diabetes |  |  |  |  |  |  |
| Q1 | 1.00 (Ref.) | 0.92 (0.86, 1.00) | 0.92 (0.85, 1.00) | 0.87 (0.79, 0.96) | 0.87 (0.74, 1.01) | 0.142 |
| Q2 | 0.94 (0.85, 1.03) | 0.82 (0.75, 0.89) | 0.84 (0.77, 0.92) | 0.83 (0.75, 0.92) | 0.85 (0.74, 0.97) |  |
| Q3 | 0.97 (0.84, 1.12) | 0.88 (0.79, 0.98) | 0.89 (0.81, 0.98) | 0.82 (0.74, 0.90) | 0.83 (0.74, 0.93) |  |
| Q4 | 0.67 (0.56, 0.82) | 0.62 (0.52, 0.74) | 0.70 (0.61, 0.80) | 0.74 (0.66, 0.83) | 0.77 (0.68, 0.87) |  |
| Q5 | 0.68 (0.55, 0.84) | 0.75 (0.61, 0.93) | 0.69 (0.56, 0.86) | 0.74 (0.61, 0.89) | 0.77 (0.66, 0.89) |  |

Hazard ratios (HRs) were estimated using Cox models with stratification on survey sites and age-at-risk (5-year groups), and adjustment for sex, age at recruitment, education level, smoking, alcohol consumption, physical activity, the average daily energy intake, spicy food, family history of CVD or diabetes, body mass index, waist circumference, prevalent diabetes, antihypertensive drugs use, and systolic blood pressure. The multiplicative interactions were tested by comparing models with and without the interaction term of two dietary patterns using the joint test.

CVD: cardiovascular disease. MCE: major coronary events. IHD: ischaemic heart disease. AMI: acute myocardial infarction. CBD: cerebrovascular disease. HS: haemorrhagic stroke. IS: ischaemic stroke.


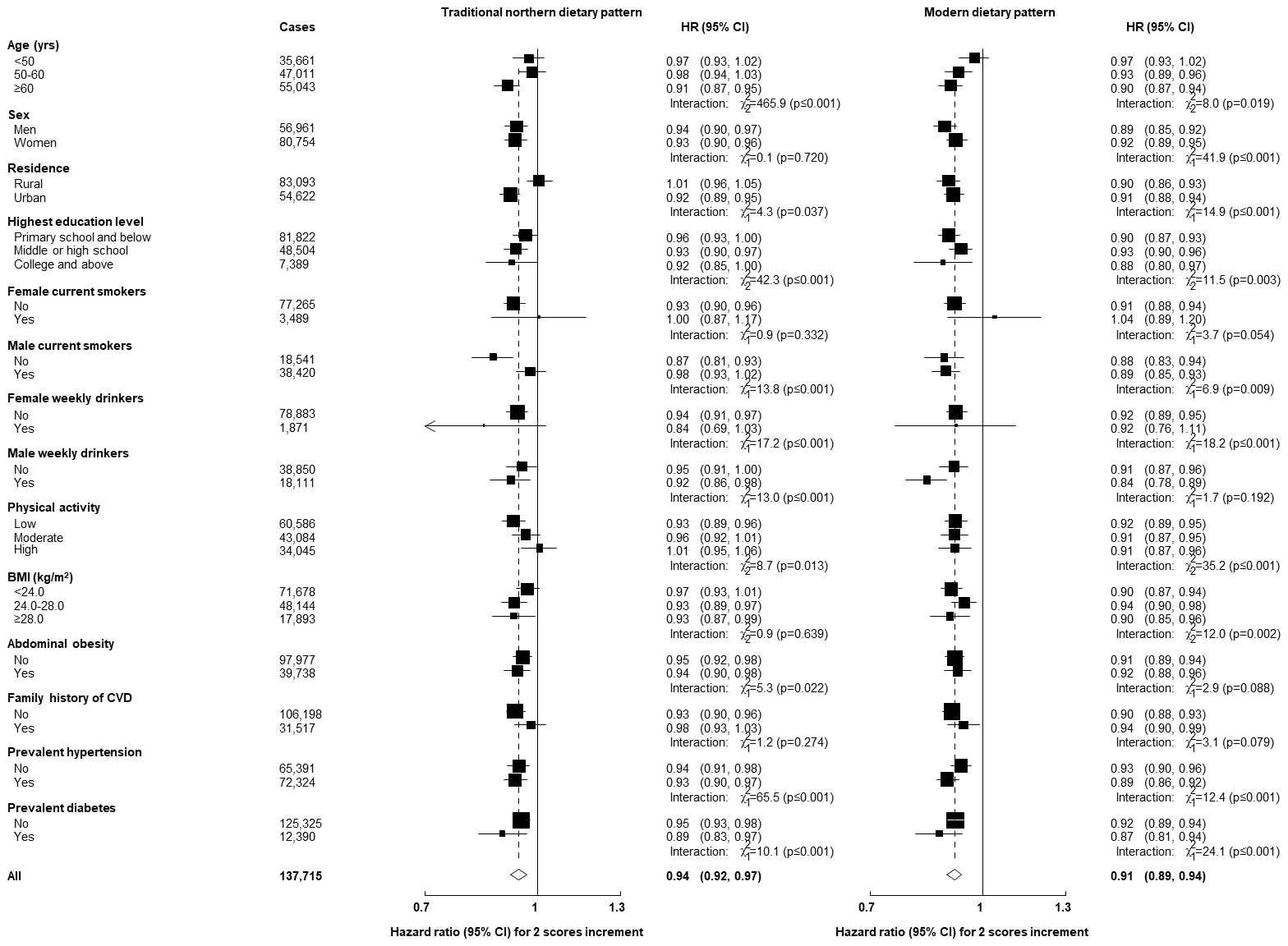


**Supplemental Figure 1 Subgroup analyses of the association between two dietary patterns and cardiovascular disease according to baseline characteristics**

Cox models were used with stratification on survey sites and age-at-risk (5-year groups), and adjusted for sex, age at recruitment, education level, smoking, alcohol consumption, physical activity, the average daily energy intake, spicy food, family history of CVD, body mass index, waist circumference, prevalent diabetes, antihypertensive drugs use, systolic blood pressure, and menopause status (only for women), as appropriate. Tests for interaction were performed by using chi-square tests to compare models with and without the interaction terms of the dietary pattern (continuous) and the potential modifier (categorical).

Abdominal obesity was defined if a man had a waist circumference ≥90cm or a woman had a waist circumference ≥85cm

BMI: body mass index.


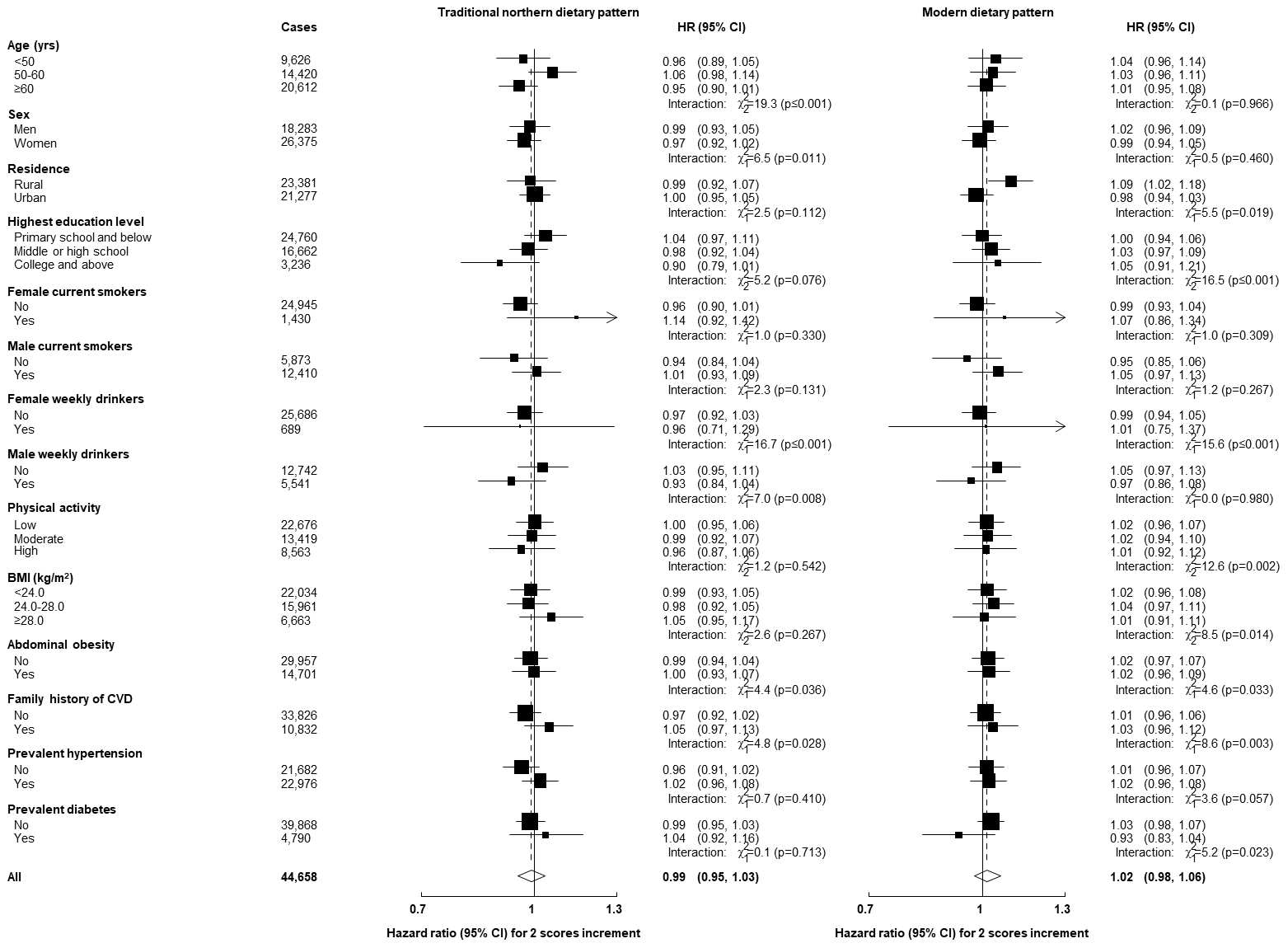


**Supplemental Figure 2 Subgroup analyses of the association between two dietary patterns and ischaemic heart disease according to baseline characteristics**

Cox models were used with stratification on survey sites and age-at-risk (5-year groups), and adjusted for sex, age at recruitment, education level, smoking, alcohol consumption, physical activity, the average daily energy intake, spicy food, family history of CVD, body mass index, waist circumference, prevalent diabetes, antihypertensive drugs use, systolic blood pressure, and menopause status (only for women), as appropriate. Tests for interaction were performed by using chi-square tests to compare models with and without the interaction terms of the dietary pattern (continuous) and the potential modifier (categorical).

Abdominal obesity was defined if a man had a waist circumference ≥90cm or a woman had a waist circumference ≥85cm

BMI: body mass index.


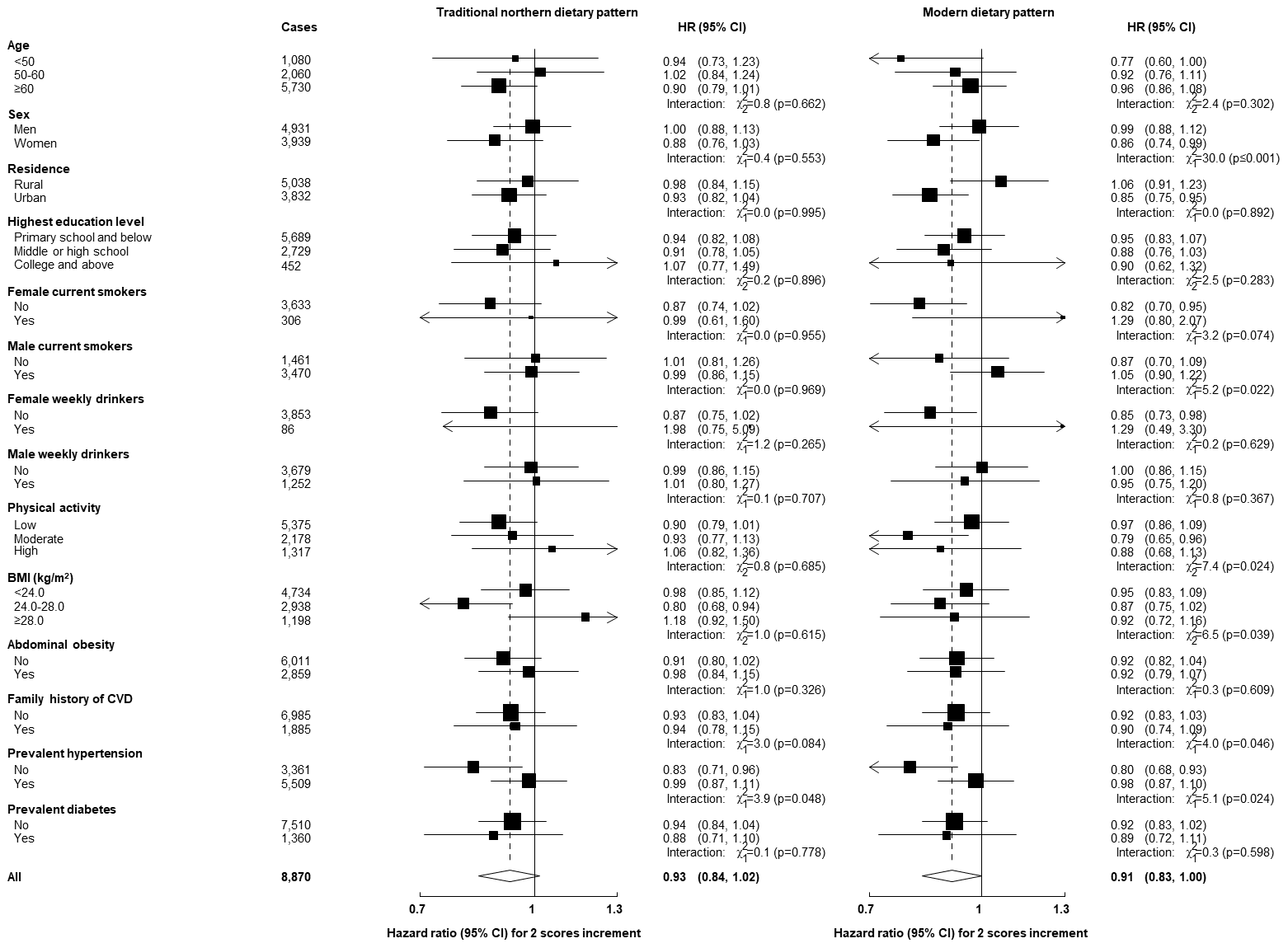


**Supplemental Figure 3 Subgroup analyses of the association between two dietary patterns and major coronary events according to baseline characteristics**

Cox models were used with stratification on survey sites and age-at-risk (5-year groups), and adjusted for sex, age at recruitment, education level, smoking, alcohol consumption, physical activity, the average daily energy intake, spicy food, family history of CVD, body mass index, waist circumference, prevalent diabetes, antihypertensive drugs use, systolic blood pressure, and menopause status (only for women), as appropriate. Tests for interaction were performed by using chi-square tests to compare models with and without the interaction terms of the dietary pattern (continuous) and the potential modifier (categorical).

Abdominal obesity was defined if a man had a waist circumference ≥90cm or a woman had a waist circumference ≥85cm

BMI: body mass index.

**
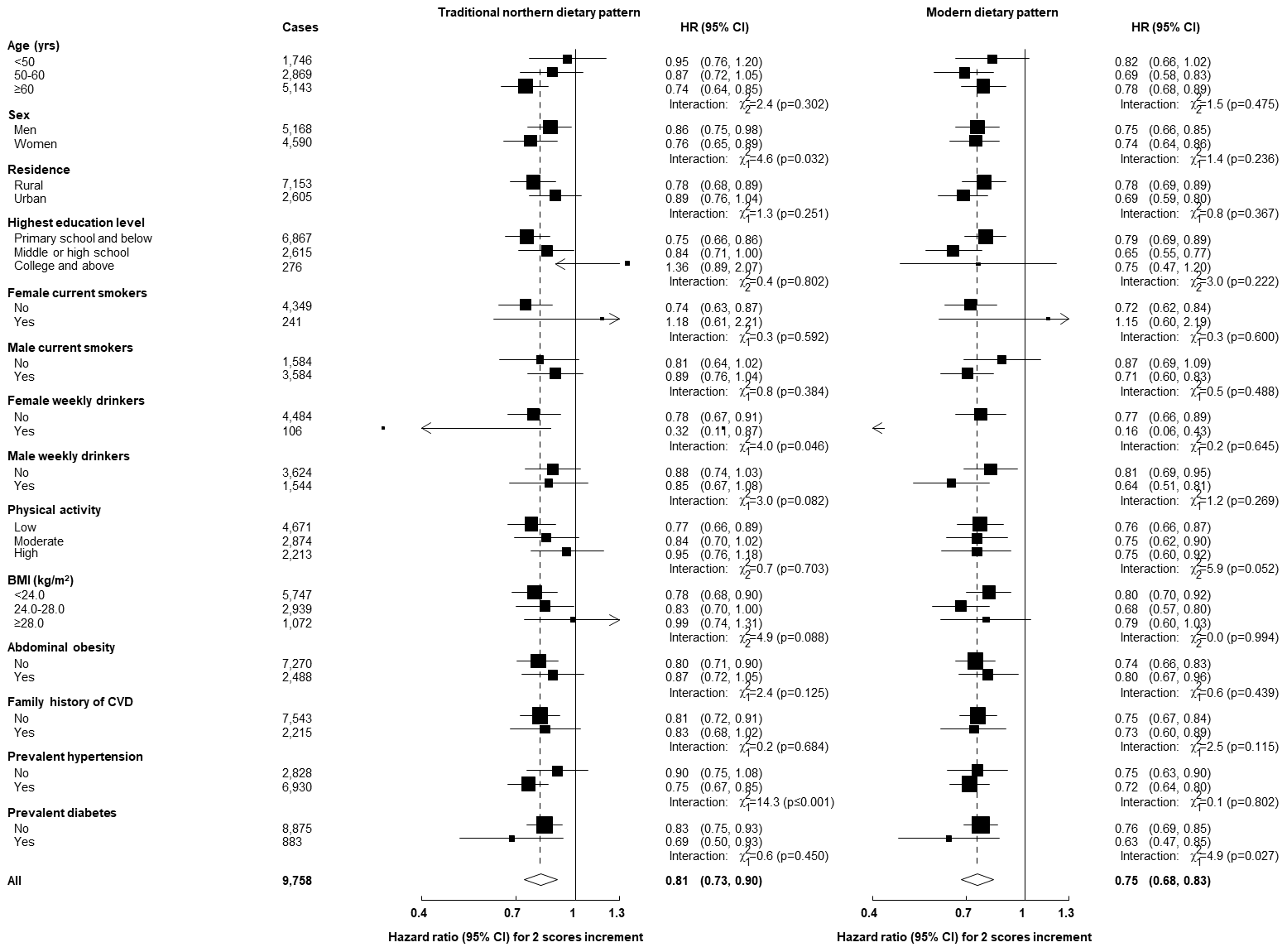
**

**Supplemental Figure 4 Subgroup analyses of the association between two dietary patterns and haemorrhagic stroke according to baseline characteristics**

Cox models were used with stratification on survey sites and age-at-risk (5-year groups), and adjusted for sex, age at recruitment, education level, smoking, alcohol consumption, physical activity, the average daily energy intake, spicy food, family history of CVD, body mass index, waist circumference, prevalent diabetes, antihypertensive drugs use, systolic blood pressure, and menopause status (only for women), as appropriate. Tests for interaction were performed by using chi-square tests to compare models with and without the interaction terms of the dietary pattern (continuous) and the potential modifier (categorical).

Abdominal obesity was defined if a man had a waist circumference ≥90cm or a woman had a waist circumference ≥85cm

BMI: body mass index.


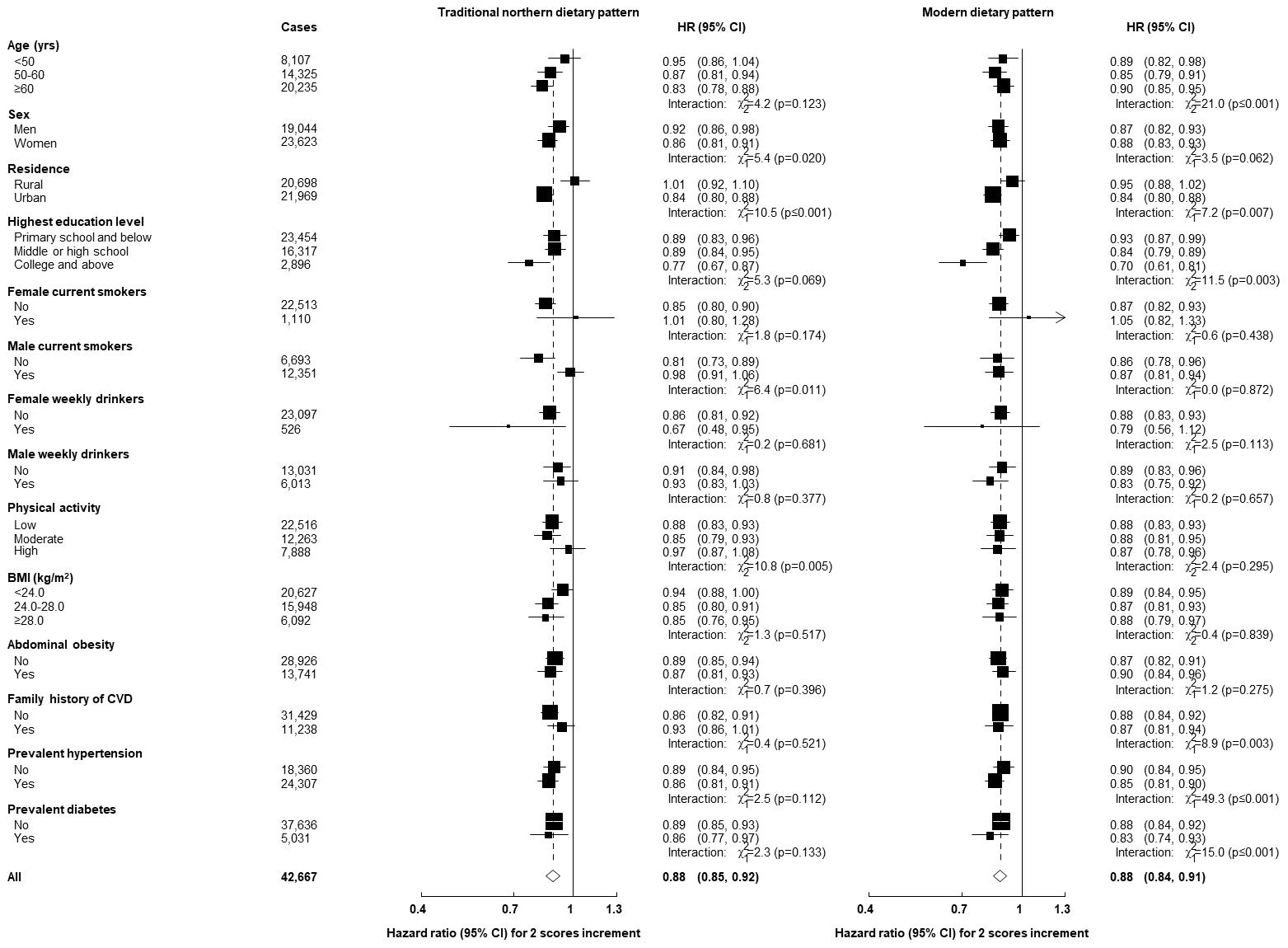


**Supplemental Figure 5 Subgroup analyses of the association between two dietary patterns and ischaemic stroke according to baseline characteristics**

Cox models were used with stratification on survey sites and age-at-risk (5-year groups), and adjusted for sex, age at recruitment, education level, smoking, alcohol consumption, physical activity, the average daily energy intake, spicy food, family history of CVD, body mass index, waist circumference, prevalent diabetes, antihypertensive drugs use, systolic blood pressure, and menopause status (only for women), as appropriate. Tests for interaction were performed by using chi-square tests to compare models with and without the interaction terms of the dietary pattern (continuous) and the potential modifier (categorical).

Abdominal obesity was defined if a man had a waist circumference ≥90cm or a woman had a waist circumference ≥85cm

BMI: body mass index.


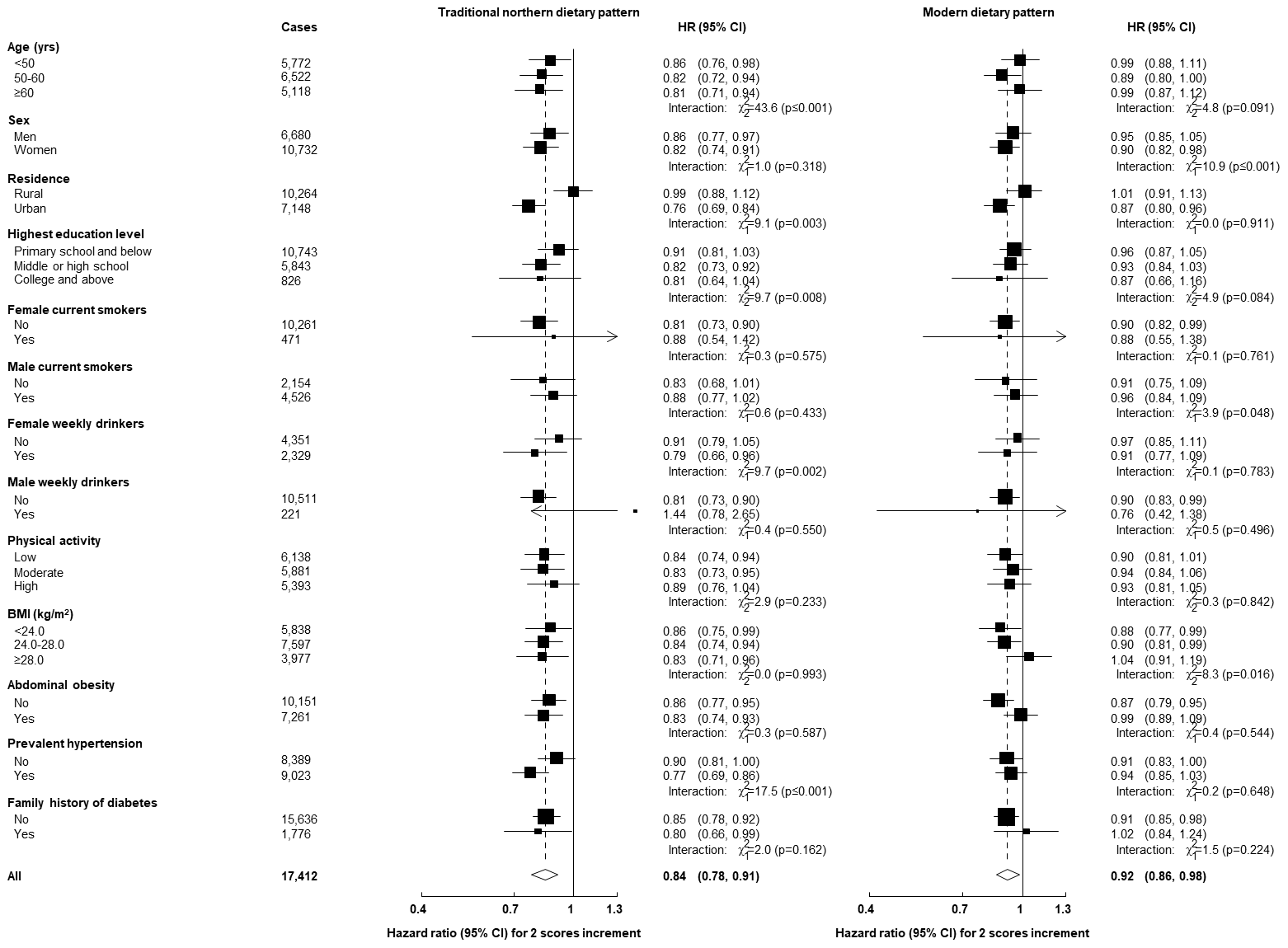


**Supplemental Figure 6 Subgroup analyses of the association between two dietary patterns and diabetes according to baseline characteristics**

Cox models were used with stratification on survey sites and age-at-risk (5-year groups), and adjusted for sex, age at recruitment, education level, smoking, alcohol consumption, physical activity, the average daily energy intake, spicy food, family history of diabetes, body mass index, waist circumference, antihypertensive drugs use, systolic blood pressure, and menopause status (only for women), as appropriate. Tests for interaction were performed by using chi-square tests to compare models with and without the interaction terms of the dietary pattern (continuous) and the potential modifier (categorical).

Abdominal obesity was defined if a man had a waist circumference ≥90cm or a woman had a waist circumference ≥85cm

BMI: body mass index.

**Members of the China Kadoorie Biobank collaborative group:**

**International Steering Committee:** Junshi Chen, Zhengming Chen (PI), Robert Clarke, Rory Collins, Yu Guo, Liming Li (PI), Jun Lv, Richard Peto, Robin Walters. **International Co-ordinating Centre, Oxford:** Daniel Avery, Ruth Boxall, Derrick Bennett, Yumei Chang, Yiping Chen, Zhengming Chen, Robert Clarke, Huaidong Du, Simon Gilbert, Alex Hacker, Mike Hill, Michael Holmes, Andri Iona, Christiana Kartsonaki, Rene Kerosi, Ling Kong, Om Kurmi, Garry Lancaster, Sarah Lewington, Kuang Lin, John McDonnell, Iona Millwood, Qunhua Nie, Jayakrishnan Radhakrishnan, Paul Ryder, Sam Sansome, Dan Schmidt, Paul Sherliker, Rajani Sohoni, Becky Stevens, Iain Turnbull, Robin Walters, Jenny Wang, Lin Wang, Neil Wright, Ling Yang, Xiaoming Yang. **National Co-ordinating Centre, Beijing:** Zheng Bian, Yu Guo, Xiao Han, Can Hou, Jun Lv, Pei Pei, Chao Liu, Canqing Yu. **10 Regional Co-ordinating Centres: Qingdao CDC:** Zengchang Pang, Ruqin Gao, Shanpeng Li, Shaojie Wang, Yongmei Liu, Ranran Du, Yajing Zang, Liang Cheng, Xiaocao Tian, Hua Zhang, Yaoming Zhai, Feng Ning, Xiaohui Sun, Feifei Li. **Licang CDC:** Silu Lv, Junzheng Wang, Wei Hou. **Heilongjiang Provincial CDC:** Mingyuan Zeng, Ge Jiang, Xue Zhou. **Nangang CDC:** Liqiu Yang, Hui He, Bo Yu, Yanjie Li, Qinai Xu,Quan Kang, Ziyan Guo. **Hainan Provincial CDC:** Dan Wang, Ximin Hu, Jinyan Chen, Yan Fu, Zhenwang Fu, Xiaohuan Wang. **Meilan CDC:** Min Weng, Zhendong Guo, Shukuan Wu,Yilei Li, Huimei Li, Zhifang Fu. **Jiangsu Provincial CDC:** Ming Wu, Yonglin Zhou, Jinyi Zhou, Ran Tao, Jie Yang, Jian Su. **Suzhou CDC:** Fang liu, Jun Zhang, Yihe Hu, Yan Lu, , Liangcai Ma, Aiyu Tang, Shuo Zhang, Jianrong Jin, Jingchao Liu. **Guangxi Provincial CDC:** Zhenzhu Tang, Naying Chen, Ying Huang. **Liuzhou CDC:** Mingqiang Li, Jinhuai Meng, Rong Pan, Qilian Jiang, Jian Lan,Yun Liu, Liuping Wei, Liyuan Zhou, Ningyu Chen Ping Wang, Fanwen Meng, Yulu Qin,, Sisi Wang. **Sichuan Provincial CDC:** Xianping Wu, Ningmei Zhang, Xiaofang Chen,Weiwei Zhou. **Pengzhou CDC:** Guojin Luo, Jianguo Li, Xiaofang Chen, Xunfu Zhong, Jiaqiu Liu, Qiang Sun. **Gansu Provincial CDC:** Pengfei Ge, Xiaolan Ren, Caixia Dong. **Maiji CDC:** Hui Zhang, Enke Mao, Xiaoping Wang, Tao Wang, Xi zhang. **Henan Provincial CDC:** Ding Zhang, Gang Zhou, Shixian Feng, Liang Chang, Lei Fan. **Huixian CDC:** Yulian Gao, Tianyou He, Huarong Sun, Pan He, Chen Hu, Xukui Zhang, Huifang Wu, Pan He. **Zhejiang Provincial CDC:** Min Yu, Ruying Hu, Hao Wang. Tongxiang CDC: Yijian Qian, Chunmei Wang, Kaixu Xie, Lingli Chen, Yidan Zhang, Dongxia Pan, Qijun Gu. **Hunan Provincial CDC:** Yuelong Huang, Biyun Chen, Li Yin, Huilin Liu, Zhongxi Fu, Qiaohua Xu. **Liuyang CDC:** Xin Xu, Hao Zhang, Huajun Long, Xianzhi Li, Libo Zhang, Zhe Qiu.
